# Geometric Direct Minimization for Low-Spin Restricted Open-Shell Hartree–Fock Theory

**DOI:** 10.1021/acs.jctc.5c00898

**Published:** 2025-09-17

**Authors:** Hugh G. A. Burton

**Affiliations:** Department of Chemistry, 150385 University College London, London WC1H 0AJ, U.K.

## Abstract

It has recently been shown that configuration state functions (CSFs) with local orbitals can provide a compact reference state for low-spin open-shell electronic structures, such as antiferromagnetic states. However, optimizing a low-spin configuration using self-consistent field (SCF) theory has been a long-standing challenge since each orbital must be an eigenfunction of a different Fock operator. We introduce a low-spin restricted open-shell Hartree–Fock (ROHF) algorithm to optimize any CSF at mean-field cost. This algorithm employs quasi-Newton Riemannian optimization on the orbital constraint manifold to provide robust convergence, extending the geometric direct minimization approach to open-shell electronic structures with arbitrary genealogical spin coupling. Numerical calculations on transition metal aquo complexes show improved convergence over existing methodology, while the possibility of local CSF energy minima is demonstrated for iron–sulfur complexes. Finally, open-shell CSFs with different spin coupling patterns are used to qualitatively study the singlet ground state in polyacenes, revealing the onset of polyradical character as the chain length increases.

## Graphical abstract

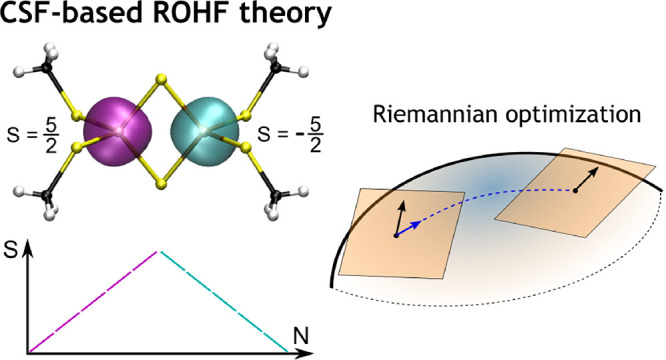

## 1 Introduction

Open-shell electronic configurations underpin quantum phenomena such as the magnetic properties of transition metal complexes,^1^ the spin-state energetics of radicals,^2^ and molecular excited states.^3^ However, theoretically characterizing open-shell states is complicated due to the near degeneracy of configurations with complex spin alignment, such as ferromagnetic and antiferromagnetic states. Developing efficient methods that accurately predict low-lying energy states of open-shell systems, while retaining conceptual understanding, remains a major challenge.

Wave function predictions of open-shell states require multiconfigurational approximations that account for the “static correlation” associated with nearly degenerate configurations. The complete active space self-consistent field (CASSCF) approach is the most common, whereby a full configuration interaction (FCI) is constructed within a set of active orbitals that are optimized simultaneously.^4,5^ However, CASSCF calculations are notoriously challenging because: the computational cost scales exponentially with the size of the active space; results are sensitive to the choice of active orbitals;^6^ and the numerical optimization can be poorly conditioned,^7–12^ with many possible stationary points.^13,14^ Furthermore, large active spaces are required to accurately compute low-lying states in polynuclear transition metal complexes or extended conjugated molecules.^15,16^ These calculations quickly become intractable for exact diagonalization and rely on approximate solvers^17^ including FCI quantum Monte Carlo (FCIQMC),^18^ density matrix renormalization group (DMRG),^16,19,20^ or selected CI.^21–25^ Even then, computing “dynamic correlation” on top of a CASSCF wave function remains a formidable challenge.

The complexity of CASSCF theory raises the question of whether alternative single-reference methods can be designed to encode the dominant static correlation and spin coupling without the need for an active space. It is well-known that a single Slater determinant can provide a good approximation to high-spin systems (with unpaired electrons all spin aligned) but is inadequate for low-spin cases.^2^ However, it has only recently been discovered that many antiferromagnetic low-spin states can be accurately represented by a small number of configuration state functions^26^ (CSF), sometimes only one.^27–30^ This approach relies on localized molecular orbitals (MOs) and an appropriate orbital ordering that combines local ferromagnetic coupling with long-range antiferromagnetic coupling.^27,30–32^ Using an optimal CSF basis can significantly reduce the multireference character of the wave function and provide a sparser representation of the Hilbert space compared to an RHF-based determinant basis.^27,30,33^ These properties have been exploited to accelerate the convergence of spin-adapted FCI solvers, such as GUGA-FCIQMC^34,35^ or selected CI,^36,37^ and to define accurate initial states for future quantum computing algorithms.^32,38^

In practice, a major challenge to using single CSF reference states for open-shell correlation theory is finding the optimal MOs through an initial Hartree–Fock (HF) calculation. While the HF equations are straightforward to solve for a closed-shell determinant,^39,40^ this becomes much harder for a low-spin open-shell configuration since each optimal spatial orbital ψ_*i*_ is an eigenfunction of a different Fock operator *f̂*_*i*_, satisfying *f̂*_*i*_ |ψ_*i*_⟩ = ϵ_*i*_ |ψ_*i*_⟩.^41^ (Note that orbitals experiencing the same Fock operator are said to occupy the same “shell”.) While several algorithms to solve the Roothaan–Hall equations for restricted open-shell HF (ROHF) have been developed,^41–47^ their generalization to low-spin CSFs with arbitrary spin coupling was only recently achieved by Neese and co-workers.^48^ However, SCF algorithms based on Fock diagonalization can be difficult to converge in the case of near degeneracies and are not guaranteed to converge to an energy minimum.

The aim of this work is to develop a quasi-Newton direct minimization algorithm for arbitrary low-spin CSF states that provides robust convergence to an energy minimum. Quasi-Newton optimization techniques can significantly improve SCF convergence in challenging cases, and have been widely adopted for HF and multiconfigurational SCF calculations.^7–12,49–59^ A particularly successful approach is Geometric Direct Minimization (GDM),^55^ which takes into account the Riemannian geometry of the orthonormal MO coefficients for a single Slater determinant. While GDM has previously been extended to high-spin ROHF calculations,^56,60^ here I introduce a general formulation for a single CSF with arbitrary spin coupling. The resulting “CSF-GDM” approach provides robust energy minimization for arbitrary low-spin CSFs, avoiding the need to handle a different Fock operator for each shell.

Developing the CSF-GDM approach provides two opportunities to further study the utility of CSF-based ROHF theory that are outlined below.

First, we currently have limited knowledge about the properties of optimal CSF solutions. For example, does the physically intuitive orbital localization and ordering actually exist as a minimum of the CSF energy? Furthermore, it is known that unrestricted HF can yield many local minima for open-shell systems, associated with localizing the unpaired electrons and breaking spin symmetry,^14,61–72^ and it is important to assess whether CSF-based ROHF is also susceptible to multiple minima. The CSF-GDM algorithm allows the electronic energy landscape^72,73^ of CSF-based ROHF theory to be systematically investigated by ensuring that calculations initialized with randomly perturbed orbital coefficients converge to a local minimum. Here, I test this approach for different spin states in model iron–sulfur clusters, revealing that many local minima can exist and that solutions with unpaired electrons localized in Fe 3d orbitals (which might be predicted from chemical intuition) are not necessarily local minima for all CSF spin states.

Second, broken-spin Kohn–Sham Density Functional Theory (KS-DFT) is currently the method of choice to qualitatively understand the electron localization in open-shell states. However, the presence of broken spin symmetry is not a perfect indicator of open-shell character since “artificial” symmetry breaking is well-documented in molecules that would normally be considered to have a closed-shell ground state.^63,66,74,75^ I show that CSF-based ROHF theory can provide qualitative insights into electron localization by comparing the energies of open-shell CSFs with different numbers of unpaired electrons, retaining both a mean-field computational cost and conserving spin symmetry. Applying this approach to the singlet ground state in polyacenes reveals the onset of polyradical character as the number of rings increases, confirming previous predictions using broken-spin KS-DFT.^76^

The remainder of this work is structured as follows. Section 2 describes the differential geometry of the ROHF wave function and energy. Although many of these expressions have been derived elsewhere, a comprehensive description is provided for reference and completeness. The CSF-GDM algorithm is then derived for arbitrary genealogical spin coupling in Section 3, with computational details in Section 4. Numerical results detailing the convergence performance of CSF-GDM, the multiple solutions for the iron–sulfur complexes [Fe­(SCH_3_)_4_]^−^ and [Fe_2_S_2_(SCH_3_)_4_]^2–^, and the open-shell character of polyacenes are described in Section 5. The primary conclusions and outlook for future work are summarized in Section 6.

## 2 Differential Geometry of the ROHF Energy

### 2.1 Definition of a Configuration State Function

A CSF corresponds to a spin-adapted linear combination of Slater determinants that is an eigenstate of both the spatial orbital number operator *n̂*_*p*_ and the total spin *Ŝ* ^2^.^26^ Here, doubly occupied closed-shell orbitals are indexed *i*, *j*, *k*, singly occupied open-shell orbitals are indexed *v*, *w*, *x*, and unoccupied orbitals are indexed *a*, *b*, *c*. Arbitrary orbitals are indexed *p*, *q*, *r*. The genealogical coupling scheme is the most common method to build a CSF, whereby open-shell electrons are sequentially coupled while maintaining an eigenstate of *Ŝ* ^2^. A particular CSF spin coupling is specified by a vector ***t***, where *t*_*i*_ denotes the change in the total spin *S* by coupling the *i*-th open-shell electron to the (*i* – 1) previous open-shell electrons. For example, $t=\left(+\frac{1}{2},-\frac{1}{2}\right)$ , denoted [+-] for convenience, corresponds to the open-shell singlet (*S* = 0)

$$\left|\Psi \rangle =\frac{1}{\sqrt{2}}|\psi_{1}\psi_{2}\rangle (|\alpha \beta \rangle -|\beta \alpha \rangle \right)$$ 1

while [++] denotes the high-spin triplet state (*S* = 1)

$$\left|\Psi \rangle =|\psi_{1}\psi_{2}\rangle |\alpha \alpha \right\rangle$$ 2

The length of ***t*** defines the number of open-shell electrons *N*_o_, with the remaining core orbitals doubly occupied, and the total spin *S* is the sum *S* = ∑ _*i* = 1_^*N*_o_^ *t*_*i*_.

The unpaired electrons can be grouped into sets of sequential + ’s or – ’s in the spin coupling vector. These groups are historically known as “shells” in ROHF theory because the electrons within each group experience the same effective 1-body Hamiltonian.^41^ For example, the singlet [+−] contains two open shells, [+] and [−], while the triplet [++] has only one open shell. Transformations that mix orbitals in the same shell leave the wave function unchanged, except for a global phase, and the doubly occupied orbitals and unoccupied virtual orbitals are considered as additional shells. Here, different shells (including the doubly occupied and unoccupied shells) are labeled with the calligraphic indices P,Q etc and the cardinality $D_{P}≡|P|$ is the number of spatial orbitals in shell P . The shell structure of the doublet configuration [++−] with the doubly occupied shell C , two singlet occupied shells P≡[++] and Q≡[−] and the unoccupied shell A is illustrated schematically in Figure 1. Crucially, a CSF is not invariant to mixing orbitals in different shells, so the wave function depends on the assignment of spatial orbitals to each shell, often referred to as the orbital ordering.^27,30^

**Figure 1:**
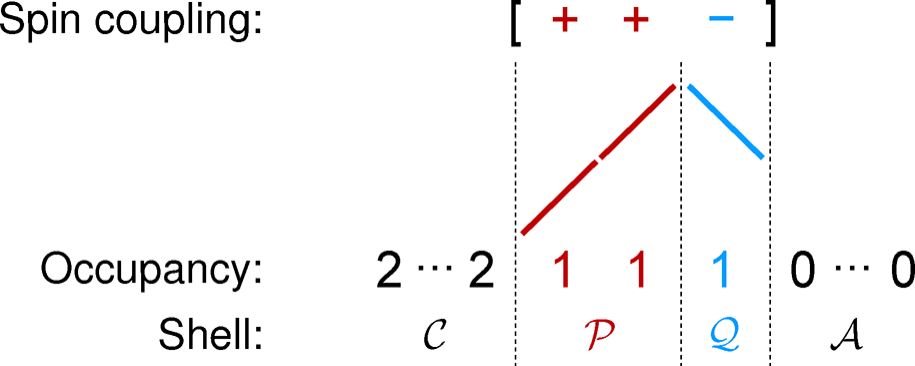
Orbital “shells” for the [++−] CSF. Each partially occupied shell is a straight-line segment in the genealogical branching diagram. The doubly occupied and unoccupied orbitals form shells C and A , respectively.

In general, there are several spin coupling vectors for each *S*, which form an orthonormal CSF basis for a fixed spatial orbital ordering. Allowed spin coupling vectors must have non-negative partial spins (∑ _*j* = 1_^*i*^ *t*_*j*_ ≥ 0 for all *i*) with $t_{1}=+\frac{1}{2}$ . The Clebsch–Gordon coupling coefficients allow any CSF to be expanded as a sum of Slater determinants with the same spatial orbital occupation and spin projection *m*_*s*_ = *S*. In practice, defining a CSF through genealogical coupling allows matrix elements to be efficiently evaluated using the unitary group approach^77–79^ (UGA) and its graphical extension^80^ (GUGA).

### 2.2 Energy of a Configuration State Function

The energy of a wave function |Ψ⟩ depends on the (partially) occupied MOs |ψ_*p*_⟩ as

$$E=\sum_{pq}h_{pq}\gamma_{pq}+\frac{1}{2}\sum_{pqrs}\Gamma_{pqrs}\langle pq|rs\rangle$$ 3

where *h*_*pq*_ denote the one-electron integrals, ⟨*pq*|*rs*⟩ are the two-electron repulsion integrals, and γ_*pq*_ and Γ_*pqrs*_ are the one- and two-electron reduced density matrices (RDMs) in the spatial MO basis. By convention, the 1- and 2-RDMs are defined in second quantization as

$$\gamma_{pq}=\langle \Psi |{\hat{E}}_{pq}|\Psi \rangle$$ 4a

$$\Gamma_{pqrs}=\langle \Psi |{\hat{e}}_{pr,qs}|\Psi \rangle$$ 4b

where the singlet excitation operator is defined as *Ê*_*pq*_ = *â*_*p↑*_^†^ *â*_*q↑*_ + *â*_*p↓*_^†^ *â*_*q↓*_, and *ê*_*pr*,*qs*_ = *Ê*_*pr*_ *Ê*_*qs*_ – δ_*rq*_ *Ê*_*ps*_.^26,77^

The spatial orbital occupancy for a single CSF must be conserved for a density matrix element to be nonzero. Therefore, the only nonzero terms are

$$\gamma_{pq}=\delta_{pq}n_{p}$$ 5a

$$\Gamma_{pqpq}=n_{p}n_{q}-\delta_{pq}n_{p}$$ 5b

$$\Gamma_{pqqp}=\langle \Psi |{\hat{E}}_{pq}{\hat{E}}_{qp}|\Psi \rangle -n_{p}$$ 5c

where *n*_*p*_ is the occupation number of spatial orbital ψ_*p*_. By defining the constants *a*_*pq*_ and *b*_*pq*_ as

$$a_{pq}≡\Gamma_{pqpq}\;\mathrm{and}\;b_{pq}≡\Gamma_{pqqp}-\delta_{pq}\Gamma_{pqpq}$$ 6

the CSF energy is then given in a similar form to Roothaan’s ROHF expression as^41^

$$E=\underset{p}{\sum }h_{pp}n_{p}+\frac{1}{2}\underset{pq}{\sum }\left(a_{pq}\left\langle pq|pq\right\rangle +b_{pq}\left\langle pq|qp\right\rangle \right)$$ 7

where the term −δ_*pq*_Γ_*pqpq*_ in the definition of *b*_*pq*_ avoids double counting of the two-electron interactions for *p* = *q*. The energy difference between CSFs with the same orbital occupation comes from only the exchange contribution ⟨Ψ|*Ê*_*pq*_ *Ê*_*qp*_|Ψ⟩. Since ⟨*pq*|*pq*⟩ = ⟨*pq*|*qp*⟩ when *p* = *q*, eq 7 is invariant to an equal and opposite shift in the diagonal constants *a*_*pp*_ and *b*_*pp*_ as long as *a*_*pp*_ + *b*_*pp*_ remains unchanged. Using ⟨Ψ |*Ê*_*pp*_ *Ê*_*pp*_| Ψ⟩ = *n*_*p*_ *n*_*p*_ then yields sufficient expressions for *a*_*pq*_ and *b*_*pq*_ as

$$a_{pq}≡n_{p}n_{q}$$ 8a

$$b_{pq}≡(1-\delta_{pq})\langle \Psi |{\hat{E}}_{pq}{\hat{E}}_{qp}|\Psi \rangle -n_{p}$$ 8b

Explicit constants for the closed-shell orbitals *i*, *j*, and open-shell orbitals *v*, *w* are found to be

$$a_{ij}=4,\;b_{ij}=-2$$ 9a

$$a_{iv}=2,\;b_{iv}=-1$$ 9b

$$a_{vw}=1,\;b_{vw}=\langle \Psi |{\hat{E}}_{vw}{\hat{E}}_{wv}|\Psi \rangle -1-\delta_{vw}$$ 9c

where *a*_*vi*_ = *a*_*iv*_ and *b*_*vi*_ = *b*_*iv*_. Here, ⟨Ψ |*Ê*_*ww*_ *Ê*_*ww*_| Ψ⟩ = 1 is used to simplify eq 8b into eq 9c. These expressions are equivalent to eqs 7−9 in ref 48, although the definition for the open-shell coupling constants differs by a factor of 2. Since all open-shell orbitals *v* in shell V and *w* in shell W share the same matrix element ⟨Ψ |*Ê*_*vw*_ *Ê*_*wv*_| Ψ⟩ = ⟨Ψ |*ê*_*vw*,*wv*_| Ψ⟩ + δ_*vw*_, the constants $b_{\mathrm{VW}}≡b_{vw}$ can be evaluated once for each pair of shells.^46,48^ Following ref 48, the *b*_*vw*_ constants can be found by using the GUGA method^80^ to derive the exchange terms ⟨Ψ |*ê*_*vw*,*wv*_| Ψ⟩ for open-shell indices *v* and *w*. An excellent derivation of these terms for a single CSF can be found in Appendix B of ref 34.

### 2.3 Analytic Gradients and Second Derivatives

The spatial MOs are parametrized in terms of *n* linearly independent atomic orbital (AO) basis functions |χ_μ_⟩ as

$$|\psi_{p}\rangle =\sum_{\mu =1}^{n}|\chi_{\mu }\rangle C_{\cdot p}^{\mu \cdot }$$ 10

where nonorthogonal tensor notation is used.^81^ The energy (eq 7) can then be minimized with respect to the orbital coefficients *C*_·*p*_^μ·^ under the orthonormality constraint

$$\sum_{\mu \nu =1}^{n}C_{p\cdot }^{\cdot \mu }\langle \chi_{\mu }|\chi_{\nu }\rangle C_{\cdot q}^{\nu \cdot }=\delta_{pq}$$ 11

where the orbital coefficients are chosen to be real, $C_{\cdot q}^{\nu \cdot }\in R$ . To satisfy orthonormality, the matrix of orbital coefficients ***C*** is constrained to a Riemannian manifold corresponding to the orthogonal group O­(*n*).^55,82^ However, since transformations between orbitals in the same shell leave the energy unchanged, only transformations between different shells should be considered as optimization parameters. The CSF optimization manifold then corresponds to the quotient space^82^

$$\frac{O(n)}{O(D_{P})\times \cdots \times O(D_{Q})}$$ 12

where each orthogonal subgroup $O(D_{P})$ represents the invariance to orthogonal transformations among the $D_{P}$ orbitals in shell P . This structure corresponds to a flag manifold, as described for high-spin ROHF in ref 60, although eq 12 provides the generalization to arbitrary low-spin open-shell configurations with more than three invariant subspaces.

The nonredundant variations of an initial CSF |Ψ_0_⟩ with orbital coefficients ***C***_0_ can be parametrized using an exponential transformation as^51^

$$\left|\Psi (\kappa )\rangle =\exp(\hat{\kappa })|\Psi_{0}\right\rangle$$ 13

where κ̂ is an anti-Hermitian one-body operator that only couples electrons in different shells as

$$\hat{\kappa }=\sum_{P\neq Q}\sum_{p\in P}\sum_{q\in Q}\kappa_{pq}({\hat{E}}_{pq}-{\hat{E}}_{qp})$$ 14

The orbital rotation step **κ** is an anti-Hermitian *n* × *n* matrix with an off-diagonal block structure, e.g., for four shells

$$\kappa =\left(\begin{array}{llll} 0 & -\kappa_{21}^{†} & -\kappa_{31}^{†} & -\kappa_{41}^{†}\\ \kappa_{21} & 0 & -\kappa_{32}^{†} & -\kappa_{42}^{†}\\ \kappa_{31} & \kappa_{32} & 0 & -\kappa_{43}^{†}\\ \kappa_{41} & \kappa_{42} & \kappa_{43} & 0 \end{array}\right)$$ 15

Since a one-body transformation corresponds to an orbital rotation, eq 13 is equivalent to the transformation

$$C(\kappa )=C_{0}\exp(\kappa )$$ 16

In practice, the orbital coefficients are updated on each iteration *k* as

$$C_{k+1}=C_{k}\exp(\kappa )$$ 17

such that the step **κ** is always expressed in the local MO basis.^55^ The advantage of parametrizing a CSF on a continuous manifold is that the assignment of orbitals to each shell is controlled by the ordering of columns in ***C***, providing more robust convergence compared to to Fock diagonalization schemes where orbitals must be allocated to shells on every iteration.^41,43,46^

Analytic gradients and second-derivatives can now be derived as a special case of multiconfigurational SCF,^51,53,83,84^ as reviewed extensively in ref 26. The gradient components are

$$g_{pq}≡\frac{\partial E}{\partial \kappa_{pq}}{|}_{\kappa =0}=2(F_{pq}-F_{qp})$$ 18

where *F*_*pq*_ are elements of the (nonsymmetric) generalized Fock matrix, defined in the MO basis as^26^

$$F_{pq}=\sum_{r=1}^{n}\gamma_{pr}h_{rq}+\sum_{rst=1}^{n}\Gamma_{prst}\langle st|qr\rangle$$ 19

Note that *F*_*aq*_ = 0 if the first index corresponds to an orbital in the unoccupied shell. The generalized Fock matrix element for cases where the first index *p* corresponds to an MO in shell P is given by

$$F_{pq}^{P}=n_{P}(h_{pq}+J_{pq})+K_{pq}^{P}$$ 20

where the superscript P indicates that the matrix element is computed using the exchange operator $K_{pq}^{P}$ experienced by shell P . The total Coulomb operator *J*_*pq*_ and the shell exchange operator $K_{pq}^{P}$ are defined as

$$J_{pq}=\sum_{r}n_{r}\langle pr|qr\rangle$$ 21a

$$K_{pq}^{P}=\sum_{R}b_{\mathrm{PR}}\sum_{r\in R}\langle pr|rq\rangle$$ 21b

Crucially, these Coulomb and exchange matrices can be evaluated using standard JK-builds in the AO basis, allowing the gradient to be obtained with $O(N_{s}n^{4})$ scaling, where *N*_s_ is the number of shells. The gradient is then given explicitly as

$$g_{pq}=2(F_{pq}^{P}-F_{qp}^{Q})$$ 22

The Hessian matrix of second derivatives,^26^ defined as

$$Q_{pq,rs}≡\frac{\partial^{2}E}{\partial \kappa_{pq}\partial \kappa_{rs}}{|}_{\kappa =0}$$ 23

can be obtained in analytic form for a single CSF as

$$\begin{array}{rl} Q_{pq,rs} & =P_{pq}P_{rs}[2\delta_{pr}F_{qs}^{P}-\delta_{qs}(F_{pr}^{P}+F_{rp}^{R})\\ & +2(2a_{pr}\langle qr|ps\rangle +b_{pr}(\langle qp|rs\rangle +\langle qr|sp\rangle ))]. \end{array}$$ 24

where the operator *P*_*pq*_ = 1 – (*pq*) introduces an antisymmetric permutation of the indices *p* and *q*. A full derivation of eq 24 is provided in Appendix A. Crucially, this expression can be used to precondition the optimization and accelerate convergence (Section 3.3).

## 3 Geometric Direct Minimization for an Arbitrary Open-Shell CSF

Section 2 introduced the necessary prerequisites to develop a Riemannian optimization algorithm for a CSF with arbitrary genealogical spin coupling. In contrast to optimization in flat Euclidean spaces, Riemannian optimization on a smooth manifold takes into account the curvature of the manifold and changes in the tangent space at different points.^82,85^ The manifold curvature means that tangent vectors must be carefully translated between points to ensure that they remain in tangent space, using a process known as parallel transport (Figure 2). Riemannian optimization accounts for this parallel transport to provide robust convergence in a curved space. Riemannian optimization based on the L-BFGS algorithm^86–89^ has previously been applied to single determinant SCF theory in the GDM algorithm,^55,56^ and has recently been extended to high-spin ROHF and CASSCF theory.^60^ Here, I extend GDM to the case of a low-spin CSF, providing robust optimization for any genealogical spin coupling and an arbitrary number of open shells.

**Figure 2:**
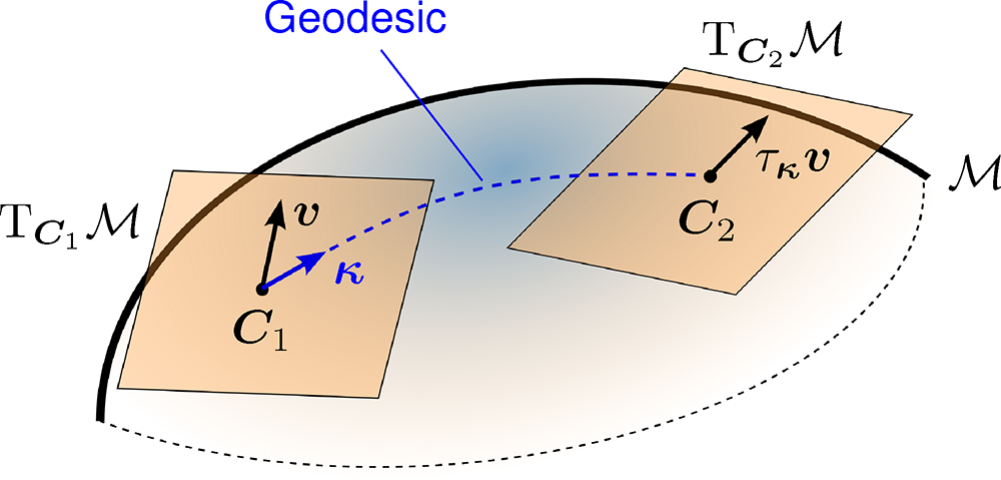
On a curved Riemannian manifold M , the tangent space $T_{C}M$ changes at each point ***C***. Tangent vectors $v\in T_{C}M$ must be parallel transported along the geodesic defined by a step direction **κ** such that the transported vector τ_**κ**_ ***v*** remains in the tangent space at the new point.

### 3.1 Quasi-Newton L-BFGS Optimization

Quasi-Newton methods use the local gradient ***g*** and steps ***s*** to estimate the inverse Hessian matrix and provide approximate second-order optimization of a multivariate function *f*(***x***).^90^ On each iteration *k*, an approximation to the inverse Hessian matrix ***B***_*k*+1_ is constructed and a downhill step ***s***_*k*+1_ is identified using the Newton–Raphson formula

$$s_{k+1}=-B_{k+1}g_{k+1}$$ 25

In the BFGS algorithm, ***B***_*k*_ is defined recursively as^86–89^

$$B_{k+1}=(I-\rho_{k}s_{k}y_{k}^{†})B_{k}(I-\rho_{k}s_{k}y_{k}^{†})+\rho_{k}s_{k}s_{k}^{†}$$ 26

where the standard definitions are

$$y_{k}=g_{k+1}-g_{k},\;s_{k}=x_{k+1}-x_{k},\;\rho_{k}=\frac{1}{y_{k}^{†}s_{k}}$$ 27

The definition of eq 26 maintains a positive definitive approximate inverse Hessian, which ensures downhill optimization steps. In the limited-memory L-BFGS variant,^91^ ***B***_*k*_ is constructed using gradients and steps from only the *m* most recent iterations, where typically *m* ∼ 10–20.^90^ Suitable choices of the initial inverse Hessian approximation ***B***_*k*+1_^0^ can significantly accelerate convergence by preconditioning the optimization (see Section 3.3).

For CSF optimization, the exponential MO parametrization means that analytic gradients can only be computed at **κ** = **0**.^51^ Therefore, instead of using global coordinates, the origin is updated on each iteration using eq 17 with the orbital rotation step **κ** = ***s***_*k*+1_, before the new gradient is computed in local coordinates corresponding to local MO–MO transformations. The local orbital rotation steps **κ** are then directly used to define ***s***_*k*_ in the L-BFGS update. An approximate line search using the Wolfe conditions^92,93^ can be used to adjust the step size to avoid understepping or overstepping, and to maintain a positive definite ***B***_*k*_.^90^

### 3.2 Parallel Transport for CSF Orbital Coefficients

Since the CSF orbital coefficients ***C*** are constrained to a curved flag manifold M , the gradient and orbital rotation step must lie in the linear tangent space $T_{C}M$ at any given point. This geometric constraint is illustrated in Figure 2. Updating the orbital coefficients corresponds to moving along the geodesic in the direction defined by the tangent step direction **κ**. The curvature of the flag manifold means that the tangent space changes at every point ***C***, and thus the gradients ***g***_*k*_ and steps ***s***_*k*_ from previous L-BFGS iterations must be parallel transported into the tangent space for the current position ***C***_*k*_ before ***B***_*k*_ is evaluated (see Figure 2). The formula to parallel transport a tangent vector $v\in T_{C}M$ on a flag manifold along the geodesic defined by **κ** has previously been derived for the high-spin ROHF case by Vidal et al.,^60^ and can be directly extended to an arbitrary CSF. Specifically, the parallel transported vector τ_**κ**_ ***v*** can be computed as

$$\tau_{\kappa }v=\sum_{k=0}^{\infty }\frac{1}{k!}{\left(-\frac{1}{2}\right)}^{k}[\kappa ,[\cdots ,[\kappa ,v]]{]}_{T}^{k}$$ 28

where [**κ**, [···, [**κ**, ***v***]]]_T_^*k*^ denotes a *k*-fold nested set of projected commutators.^60,94^ Each individual commutator [**κ**, ***v***]_T_ denotes a standard matrix commutator [**κ**, ***v***] projected into the tangent space T such that the components of [**κ**, ***v***] that connect two orbitals in the same shell are removed and [**κ**, ***v***]_T_ adopts the block off-diagonal structure shown in eq 15. The cost to evaluate [**κ**, ***v***]_T_ scales as $O(n^{3})$ . In practice, eq 28 can be evaluated recursively and truncated when the maximum element in the highest-order nested projected commutator falls below a threshold value. Since the orbital rotation step **κ** is generally small, this recursive scheme typically converges in at most 5 steps with a threshold of 10^–4^. Therefore, the cost of parallel transport is negligible compared to evaluating the Coulomb and exchange matrices.

If the CSF is a closed-shell determinant, then the orbital coefficients are constrained to a Grassmann manifold,^55^ which is a special case of a flag manifold with only two invariant subspaces (the occupied and virtual shells). In that case, eq 28 leaves tangent vectors ***v*** unchanged and the inverse Hessian update can be performed directly using ***s***_*k*_ and ***g***_*k*_ from previous iterations without parallel transport.^55^ However, this property is no longer satisfied for an open-shell CSF, and thus the previous steps and gradients required for the L-BFGS update should be parallel transported on every iteration.

### 3.3 Preconditioner and Energy-Weighted Coordinates

The convergence of quasi-Newton algorithms can be significantly accelerated by using a preconditioner to ensure that the approximate inverse Hessian closely approximates the identify matrix. For the CSF-GDM optimization, two methods are combined to achieve successful preconditioning. First, the initial inverse Hessian is defined as a scaled identity matrix^90^

$$B_{k+1}^{0}=\gamma_{k+1}I$$ 29

where γ_*k*_ approximates the local curvature as

$$\gamma_{k+1}=\frac{s_{k}^{†}y_{k}}{y_{k}^{†}y_{k}}$$ 30

Second, a transformation into pseudocanonical energy-weighted coordinates (EWCs) is used to construct a local coordinate system for the tangent space that approximately diagonalizes the Hessian.^55,95^

Constructing pseudocanonical EWCs requires an approximation to the diagonal elements of the Hessian *Q*_*pq*,*pq*_. From the analytic expression (eq 24), the diagonal terms are given exactly as^53^

$$\begin{array}{rl} Q_{pq,pq} & =2(F_{qq}^{P}-F_{qq}^{Q})+2(F_{pp}^{Q}-F_{pp}^{P})\\ & +4(a_{pp}+a_{qq}-4a_{pq})\langle pq|qp\rangle \\ & +2(b_{pp}+b_{qq}-2a_{pq})(\langle pq|qp\rangle +\langle pq|pq\rangle ). \end{array}$$ 31

Following, ref 53, the explicit two-electron integrals must be included for *Q*_*wq*,*wq*_ when *w* is an open-shell orbital, since the energy change for transformations involving open-shell orbitals can be small. The relevant Coulomb and exchange integrals ⟨*wq*|*wq*⟩ and ⟨*wq*|*qw*⟩ can be evaluated using a *J* and *K* matrix build for each open-shell orbital with overall scaling $O(N_{o}n^{4})$ . These two-electron integrals can be ignored for core–virtual rotations, giving

$$Q_{ia,ia}\approx 2(F_{aa}^{C}-F_{aa}^{A})+2(F_{ii}^{A}-F_{ii}^{C})$$ 32

where C ( A ) denotes the core (virtual) orbital shell.

Two transformations can now be performed to bring the Hessian into an approximate diagonal form. The first is to construct pseudocanonical orbitals by transforming the orbitals within each invariant subspace (orbital shell) to diagonalize the generalized Fock matrix within that subspace, i.e., to bring $F_{pq}^{P}$ into a diagonal form for p,q∈P . This canonical transformation can be achieved using an orthogonal transformation ***C*** → ***C*** ***U*** with block diagonal form, e.g., for a CSF with four shells

$$U=\left(\begin{array}{llll} U_{1} & 0 & 0 & 0\\ 0 & U_{2} & 0 & 0\\ 0 & 0 & U_{3} & 0\\ 0 & 0 & 0 & U_{4} \end{array}\right)$$ 33

Since the generalized Fock matrix is zero for orbitals in the virtual shell, the corresponding invariant transformation must be approximated by diagonalizing the standard Fock matrix in the virtual–virtual subspace

$$f_{ab}=h_{ab}+\sum_{r}n_{r}\left(\left\langle ar|br\rangle -\frac{1}{2}\langle ar|rb\right\rangle \right)$$ 34

In the second transformation, the tangent space coordinates are rescaled with a preconditioner

$$\alpha_{pq}=\max(|Q_{pq,pq}{|}^{1/2},t_{\alpha })$$ 35

built from *Q*_*pq*,*pq*_ in the pseudocanonical orbital basis (eqs 31 and 32) such that the approximate inverse Hessian becomes close to the identity matrix. These transformations can be combined by transforming the gradient and orbital rotation steps from previous iterations as

$$s_{k}\to U^{†}s_{k}U\;\mathrm{and}\;g_{k}\to U^{†}g_{k}U$$ 36

and then rescaling the step and gradient components as

$${\tilde{s}}_{pq}=\frac{s_{pq}}{\alpha_{pq}}\;\mathrm{and}\;{\tilde{g}}_{pq}=\alpha_{pq}g_{pq}$$ 37

In eq 35, the use of positive definite α_*pq*_ enforces downhill step directions, while the threshold *t*_α_ (set here as 0.1) prevents step components from blowing up in eq 37. Once the quasi-Newton step has been evaluated, the new orbital rotation step ***s***_*k*+1_ in the pseudocanonical basis is computed from the energy-weighted coordinates as

$$s_{pq}=\alpha_{pq}{\tilde{s}}_{pq}$$ 38

Preliminary tests showed that transforming to EWCs through eq 37 is essential to achieve satisfactory convergence and should be performed on every iteration, whereas pseudocanonicalization using eq 36 can be performed intermittently.

### 3.4 Choice of Initial Orbitals

CSF-based ROHF theory can strongly depend on the choice of initial orbitals for certain spin coupling patterns, as shown by the numerical results in Section 5 (vide infra) and in ref 48. In this work, two strategies are considered to identify the initial orbital coefficients. The first is to initialize the coefficients by performing random orbital rotations from the optimized high-spin ROHF solution, allowing multiple CSF solutions to be searched. The second is to localize the high-spin ROHF orbitals using the Pipek–Mezey method^96^ (or another localization scheme) and then assign these to the open shells such that the CSF energy is minimized (Appendix B). Alternative starting guess orbitals described in ref 48 make use of the atomic valence active space^97^ procedure and spin-averaged HF,^98^ but these are not considered here.

### 3.5 Outline of the CSF-GDM Algorithm

Bringing together these components now allows the CSF-GDM algorithm to be defined as follows. Starting from some suitable initial orbital coefficients, each iteration proceeds as

1. Pseudocanonicalize MOs and update previous steps {***s***_*k*_} and gradients {***g***_*k*_} using eq 36;
2. Compute gradient ***g***_*k*+1_ using the pseudocanonical orbitals;
3. Convert all {***s***_*k*_} and {***g***_*k*_} to energy-weighted coordinates using eq 37;
4. Compute the initial inverse Hessian ***B***_*k*+1_^0^ using eq 29;
5. Compute the quasi-Newton step ***s̃***_*k*+1_ using the standard L-BFGS update formula;
6. Convert ***s̃***_*k*+1_ to an orbital rotation using eq 38;
7. Update the MO coefficients using eq 16 with **κ** = ***s***_*k*+1_ and compute the new energy *E*_*k*+1_;
8. Parallel transport previous steps {***s***_*k*_} and gradients {***g***_*k*_} to the new origin using eq 28;
9. If not converged, return to step 1.

The overall computational scaling is given by $O(N_{o}n^{4})$ , which is determined by the cost of computing the Coulomb and exchange integrals in the preconditioner eq 31. All required integrals can be computed using standard routines to compute Coulomb and exchange matrices, meaning that the algorithm can benefit from existing highly optimized Fock-build routines.

## 4 Computational Details

A pilot version of the CSF-GDM algorithm has been implemented in the Quantel library,^99^ a Python/C++ package for developing electronic structure algorithms. An interface to the PySCF package^100^ is used to evaluate all AO integrals and to construct the necessary Coulomb and exchange matrices. Optimization steps are rescaled to satisfy the criterion ∥ ***s*** ∥_*∞*_ ≤ 0.5 and the maximum number of previous steps in the L-BFGS update is 20. CSF-GDM calculations were converged to the criteria ∥ ***g*** ∥_*∞*_ ≤ 10^–6^ *E*_h_. Benchmark CSF-ROHF calculations were performed in ORCA 6.0 using the comparable VeryTightSCF convergence criteria.^101^ The maximum number of iterations for all calculations was 1000.

## 5 Results

### 5.1 Convergence Performance for Transition-Metal Compounds

#### 5.1.1 Mononuclear Hexa-Aquo Complexes

To assess the performance of the CSF-GDM algorithm, optimization statistics are compared against the recently introduced CSF-ROHF approach.^48^ The test set includes the different open-shell spin coupling states for the 3d transition metal hexa-aquo complexes with varying oxidation states, as listed in Table 1, using the def2-SVP basis set.^102^ The geometry of each complex is optimized for the high-spin state in the oxidation state for which the t_2g_ or e_g_ crystal field orbitals have equal occupation to prevent any Jahn–Teller distortion. Details of geometry optimization, and the corresponding structures, are provided in Supporting Information Section S1. The same structure is then used for all oxidation states with the same metal center. The importance of parallel transport and pseudocanonicalization is considered by performing CSF-GDM without these features, labeled as “no PT” and “no PC” respectively. If no parallel transport or pseudocanonicalization are included, then the CSF-GDM approach reduces to standard L-BFGS.

**Table 1:** Spin Coupling Vectors Considered for the 3d Transition Metal Hexa-Aquo Complexes^a^

complex	singlet	doublet	triplet	quartet
[V(H_2_O)_6_]^2+^		+ + –		
		+ – +		
[V(H_2_O)_6_]^3+^	+ –			
[Cr(H_2_O)_6_]^2+^	+ + – –		+ + + –	
	+ – + –		+ + – +	
			+ – + +	
[Cr(H_2_O)_6_]^3+^		+ + –		
		+ – +		
[Mn(H_2_O)_6_]^2+^		+ + + – –		+ + + + –
		+ + – + –		+ + + – +
		+ – + + –		+ + – + +
		+ + – −+		+ – + + +
		+ – + – +		
[Fe(H_2_O)_6_]^2+^	+ + – –		+ + + –	
	+ – + –		+ + – +	
			+ – + +	
[Fe(H_2_O)_6_]^3+^		+ + + – –		+ + + + –
		+ + – + –		+ + + – +
		+ – + + –		+ + – + +
		+ + – −+		+ – + + +
		+ – + – +		
[Ni(H_2_O)_6_]^2+^	+ –			

a 34 different spin coupling vectors and complexes are considered in total.

Statistics for the mean, median, minimum, and maximum number of iterations required to reach convergence starting from the high-spin ROHF orbitals are presented in Table 2. All the CSF-GDM variants converge in fewer iterations than the CSF-ROHF algorithm, while the latter fails to converge to the lowest energy solution in 23 out of 34 cases. The optimization is deemed to have converged to a local minimum if it does not find the lowest energy solution found across all algorithms, with a threshold of 1 μE_h_ used to identify equivalent local minima. CSF-GDM generally converges in slightly fewer iterations when both pseudocanonicalization and parallel transport are included, although this effect is relatively small. Converged CSF energies are tabulated in Supporting Information Table S1.

**Table 2:** Optimization Statistics for the 3d Transition-Metal Hexa-Aquo Complexes (see Table 1) Starting from the High-Spin ROHF Orbitals

algorithm	mean	median	min	max	local minima	fail
CSF-GDM	42.4	45.0	9	104	0	0
CSF-GDM (no PC)	43.9	47.0	9	104	1	0
CSF-GDM (no PT)	44.1	46.0	9	102	1	0
L-BFGS	44.8	49.0	9	101	0	0
CSF-ROHF	80.6	45.0	13	940	23	2

It is surprising that pseudocanonicalization and parallel transport only slightly improve the convergence behavior, even though they more accurately account for the curvature of the ROHF manifold. One possibility is that these algorithmic components are more significant when the optimization starts further away from convergence. To test this hypothesis, convergence statistics were also obtained using a core orbital guess, which is expected to be a worse starting point than the high-spin ROHF orbitals (Table 3). Converged CSF energies are tabulated in Supporting Information Table S2. Including both pseudocanonicalization and parallel transport now reduces the mean number of iterations by 7% compared to standard L-BFGS, and leads to fewer local minima. Furthermore, CSF-ROHF becomes much less robust for these less accurate starting guesses, failing to converge nine times, and leading to more iterations and local minima than the CSF-GDM approach. These results demonstrate the advantage of quasi-Newton optimization methods for CSF-based ROHF calculations, and the importance of a good initial guess.

**Table 3:** Optimization Statistics for the 3d Transition-Metal Hexa-Aquo Complexes (see Table 1) Starting from a Core Orbital Guess

algorithm	mean	median	min	max	local minima	fail
CSF-GDM	119.6	118.5	90	167	10	0
CSF-GDM (no PC)	132.4	127.0	89	221	12	0
CSF-GDM (no PT)	123.4	120.0	93	163	12	0
L-BFGS	128.5	131.0	86	170	18	0
CSF-ROHF	144.3	100.0	22	437	23	9

#### 5.1.2 Benchmark Cr_2_ and CrC Molecules

Next, the convergence performance of CSF-GDM is tested for the Cr_2_ and CrC diatomic molecules, which have become challenging benchmarks for new SCF optimization algorithms.^59,103^ Following ref 59, the def2-TZVPP basis set^102^ is used with a bond length of 2Å for both molecules. The number of iterations (*N*_Iter_) required to converge various high- and low-spin configurations with different initial guesses is reported in Table 4.

**Table 4:** Comparison of CSF-GDM and CSF-ROHF for Cr_2_ and CrC at 2.0Å Bond Length with Selected CSFs, Using the Core or High-Spin ROHF Initial Guess^a^

			CSF-GDM		CSF-ROHF (ORCA 6.0)^101^	
	spin coupling	initial guess	*N*_Iter_	*E*_min_/ *E*_h_	*N*_Iter_	*E*_min_/ *E*_h_
Cr_2_	RHF	core	209	–2086.159 612	fail	–*2085.872 496*
	[++]	core	198	–2086.213 166	29	–2085.876 204
	[+ + + +]	core	206	–2086.260 779	73	–2085.921 253
	[+ + + + + +]	core	169	–2086.309 706	38	–2085.882 240
	[+ + + + + + + +]	core	130	–2086.426 127	617	–2086.075 782
	[+ + + + + + + + + +]	core	75	–2086.527 987	30	–2086.366 815
	[+ + + + + – −––−]	core	127	–2086.177 303	fail	*–2085.917 024*
	[+ + + + + – −––−]	ROHF	43	–2086.506 284		
	[+ + + + + – −––−]	ordered ROHF	12	–2086.506 284		
CrC	RHF	core	36	–1080.566 167	52	–1080.698 666
	[++]	core	165	–1080.822 232	70	–1080.787 931
	[+ + + +]	core	139	–1080.880 312	269	–1080.902 882
	[+ + + + + +]	core	131	–1080.967 996	136	–1080.967 996
	[+ + + + + + + +]	core	98	–1081.061 727	71	–1081.061 727
	[+ + + + – −–−]	core	115	–1080.855 416	234	–1080.819 732
	[+ + + + – −–−]	ROHF	26	–1081.005 627		
	[+ + + + – −–−]	ordered ROHF	12	–1081.005 627		

a When a calculation failed to converge in 1000 iterations, the energy of the last iteration is reported in italics.

For Cr_2_, the convergence of the closed-shell RHF state using CSF-GDM in 203 iterations is competitive with other recent second-order SCF algorithms, such as the Quasi-Newton Unitary Optimization with Trust-Region (QUOTR) algorithm, which converges to the same solution in 193 iterations.^59^ In contrast, the CSF-ROHF algorithm in ORCA 6.0, which uses standard DIIS optimization, failed to locate an RHF solution, although more sophisticated algorithms, such as the trust-radius augmented Hessian method,^104^ were successful. For each high-spin ROHF configuration, CSF-GDM finds a lower energy solution than CSF-ROHF. The advantage of using localized high-spin ROHF initial orbitals that are ordered to minimize the CSF energy is shown by the rapid convergence of the antiferromagnetic [+++++––––−] CSF in 12 iterations, compared to 43 iterations using the canonical high-spin ROHF orbitals (the core initial guess converged to a local minimum). By comparison, CSF-ROHF failed to converge for this antiferromagnetic CSF starting from the core orbital guess.

Similar performance is observed for the CrC molecule, where now the low-spin ROHF convergence is demonstrated with the [++++–––−] spin coupling. CSF-GDM converges in only 36 iterations for the closed-shell RHF state, but finds a higher energy local minimum compared to ref 59 and the solution obtained from ORCA 6.0. Again, using localized and optimally ordered high-spin ROHF orbitals as the initial guess leads to a lower energy antiferromagnetic CSF compared to the core orbital guess, and converges in half as many iterations compared to starting from the canonical high-spin ROHF orbitals. In contrast, CSF-ROHF requires 234 iterations to converge for the antiferromagnetic CSF state using the core orbital guess, and finds a higher-energy solution.

### 5.2 Multiple Solutions in Iron–Sulfur Complexes

It is well-known that single determinant methods exhibit local minima, particularly when a single determinant is used to describe a multiconfigurational state. Since there is only a small change in the CSF energy when the assignment of open-shell orbitals to different shells is varied, it is likely that more local minima occur when there are many singly occupied shells. The ability to systematically converge minima from various starting points using CSF-GDM provides an opportunity to ask: what is the nature and frequency of local minima in low-spin ROHF theory? This question is investigated using the synthetic iron–sulfur complexes [Fe­(SCH_3_)_4_]^−^ and [Fe_2_S_2_(SCH_3_)_4_]^2–^, which provide models for iron–sulfur clusters involved in biological redox processes and electron transfer.^15,105^ Previous CSF energies for [Fe­(SCH_3_)_4_]^−^ with various spin coupling patterns are available from the literature for verification.^48^ Geometries for both clusters were the same as ref 48 (originally from refs 106,107 ) and the def2-TZVP basis set^102^ was used.

#### 5.2.1 Mononuclear [Fe­(SCH_3_)_4_]^−^

For the mononuclear [Fe­(SCH_3_)_4_]^−^ complex, 100 independent CSF-GDM calculations were performed starting from different initial orbital coefficients. These guesses were obtained by first converging the corresponding high-spin ROHF orbital coefficients and then applying a random orbital rotation. This process was repeated for all spin coupling patterns with five unpaired electrons and the resulting local minima energies are shown in Figure 3A. With only 100 initial guesses, these data are unlikely to provide an exhaustive set of solutions, but they are sufficient to illustrate the key properties of different local minima. Degenerate solutions are counted as one minimum in the following analysis.

**Figure 3:**
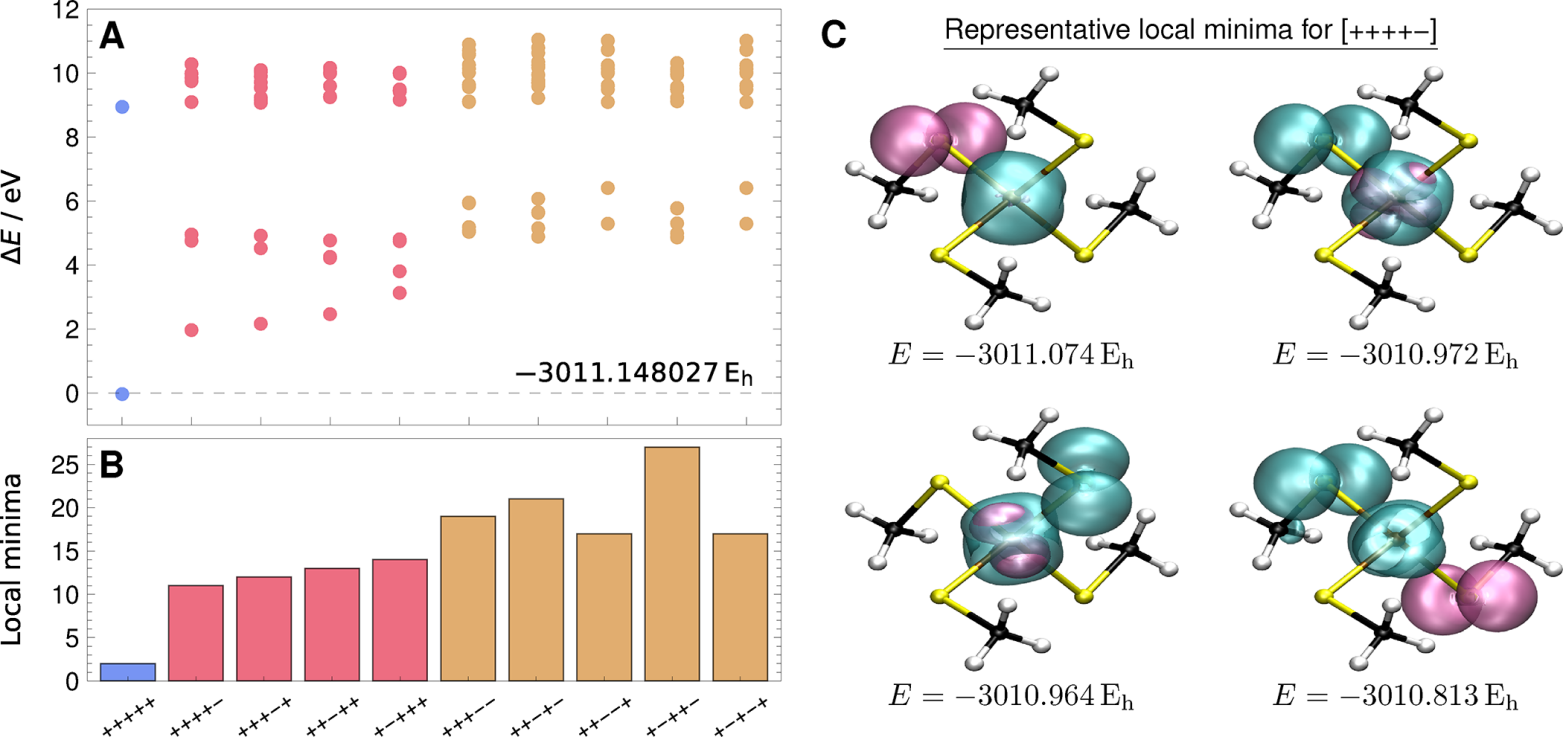
(A) Energies of the lowest local minima found through random searches for each spin-coupling pattern in the [Fe­(SCH_3_)_4_]^−^ complex (def2-TZVP), shown relative to the *S* = 5/2 ROHF global minimum. Energies are tabulated in Supporting Information Table S3. (B) Local minima generally become more prevalent as the number of unique open shells increases. (C) For the [++++−] global minimum (*E* = – 3011.074 *E*_h_), the [−] shell density (purple) is localized on a S atom while the [++++] shell density (cyan) is localized on the Fe center (top left). Higher-energy local minima correspond the different arrangements of electrons localized on one or two S atoms.

Only two minima are found for the high-spin ROHF calculation, with the lowest energy solution matching the result from ref 48. The electronic structure of the global minimum corresponds to localizing all five unpaired electrons on the Fe atom. In contrast, for the higher-energy local minimum, one unpaired electron is localized on a S atom.

For the spin-coupling patterns with *S* = 3/2, the local minima roughly form three energy groupings. The corresponding open-shell structure can be visualized by plotting the electron density for each shell of unpaired electrons, as illustrated by the density plots for the [++++−] spin coupling in Figure 3C. In the lowest energy solution (*E* = −3011.074 *E*_h_), the [−] shell (purple) is localized on a S atom, with the remaining unpaired electrons localized on the Fe center. This arrangement minimizes the repulsive exchange interaction between electrons in the [++++] and [−] shells. The next highest pair of solutions (*E* = −3010.972 *E*_h_ and *E* = −3010.964 *E*_h_) correspond to configurations where the electron in the [−] shell is localized on the Fe, with one of the [++++] electrons localized on a S atom and the remainder on Fe. Finally, in the highest-energy group of local minima, there are two unpaired electrons localized on different S atoms, as illustrated by the solution with energy *E* = −3010.813 *E*_h_ in Figure 3C. Similar patterns of electron localization are observed for the other *S* = 3/2 spin-coupling patterns, and also for the CSFs with *S* = 1/2.

The presence of several local minima with unpaired electrons localized on S atoms hints at important electron correlation processes in this complex. It is clear from these data, and previous results,^48^ that the non-Hund ordering of spin states in [Fe­(SCH_3_)_4_]^−^ is incorrectly described at the ROHF level of theory. The dominant correlation mechanisms that stabilize the low-spin states below the high-spin states are known to involve metal-to-ligand charge transfer, leading to partial open-shell character on the S atoms.^1^ This process manifests in CSF-based ROHF theory as low-energy minima with an unpaired electron in a sulfur 3p orbital, as shown in Figure 3C. The existence of local CSF minima that mimic important correlation mechanisms is analogous to symmetry-broken unrestricted HF solutions that are found for strongly correlated open-shell molecules,^71,72,108^ and has also been observed for multiple CASSCF solutions.^13,14^

These results strongly indicate that CSF-based ROHF calculations using complex spin-coupling patterns are highly susceptible to local minima. Therefore, the success of this approach relies on selecting a good initial guess that incorporates the expected electron localization. However, even if a good initial guess can be found, there is no guarantee that the expected electron configuration will form a local minimum of the CSF energy. For example, despite initializing the open-shell orbitals using localized Fe 3d orbitals obtained from a high-spin ROHF calculation, no local minimum was found with all the unpaired electrons localized on the Fe atom for any of the *S* = 3/2 or *S* = 1/2 spin-coupling patterns. This result suggests that the *S* = 3/2 or *S* = 1/2 solutions identified using CSF-ROHF in ref 48 are saddle points of the CSF energy, which can be verified using stability analysis.

#### 5.2.2 Bimetallic [Fe_2_S_2_(SCH_3_)_4_]^2–^

Next, the [Fe_2_S_2_(SCH_3_)_4_]^2–^ complex is considered as an example of a bimetallic cluster with an antiferromagnetic ground state.^15^ This larger cluster provides an opportunity to study the number of solutions as the number of open-shell electrons increases. The ferromagnetic high-spin *S* = 5 state can approximated by a CSF with the spin-coupling pattern [++++++++++], while the antiferromagnetic low-spin *S* = 0 state can be approximated by the spin-coupling pattern [+++++––––−] with the [+++++] shell localized on one Fe center and the [−–––−] shell on the other Fe. It was previously shown that these CSF approximations alone do not predict the correct energetic ordering of the two spin states.^48^ Correctly predicting the antiferromagnetic ground state requires additional electron correlation mechanisms involving charge transfer and ionic configurations,^1^ which are not present in the CSF approximation.

Following the same procedure as the mononuclear complex, 100 independent CSF-GDM calculations were performed using different starting points obtained through a random perturbation to the lowest-energy high-spin (*S* = 5) orbital coefficients. The resulting local minima are plotted for the two spin states in Figure 4A, with energies shown relative to the lowest-energy high-spin solution (*E* = – 5068.259547 *E*_h_). Notably, for the low-spin CSF, the expected antiferromagnetic Fe–Fe coupling was not found using any of the 100 random starting points. This surprising result indicates that the CSF energy landscape can have many local minima with small catchment basins, emphasizing the dependence on the initial guess. Instead, a physically motivated guess was defined by first converging the lowest-energy high-spin solution, then localizing the open-shell orbitals, and finally assigning these local orbitals. to each shell in an order that minimizes the exchange interaction, using the algorithms described in Appendix B. The resulting solution was found to be the lowest energy antiferromagnetic CSF with the two open shells centered on separate Fe atoms (Figure 4C, bottom), and is nearly degenerate with the *S* = 5 global minimum (Figure 4B, bottom).

**Figure 4:**
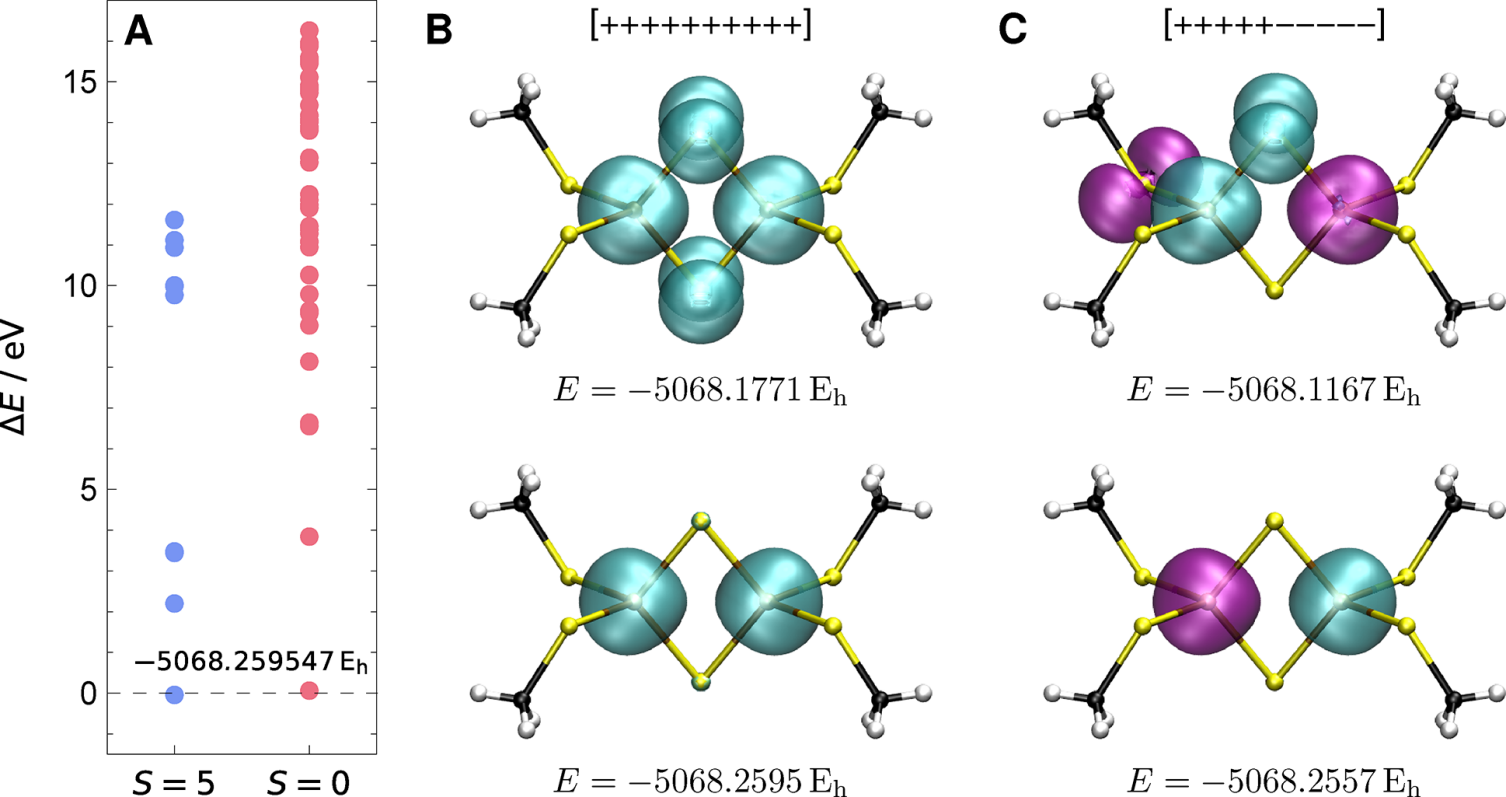
(A) Energies of the lowest local minima for the *S* = 5 ferromagnetic [++++++++++] and *S* = 0 antiferromagnetic [+++++–––−] spin coupling in [Fe_2_S_2_(SCH_3_)_4_]^2–^ (def2-TZVP) relative to the *S* = 5 global minimum. Converged energies are tabulated in Supporting Information Table S4. (B) Singly occupied shell density plots for the two lowest energy *S* = 5 minima show that the global minimum (bottom) only has unpaired electrons on Fe atoms, while the first local minimum (top) has open-shell character on S atoms. (C) Singly occupied shell density plots for the two lowest energy *S* = 0 minima show that the global minimum (bottom) has the [+++++] shell (cyan) localized on one Fe and the [−–––−] shell (purple) on the other Fe. Again, the first local minimum (top) has unpaired electrons on S atoms.

The lowest-energy solution for both spin states corresponds to the ferromagnetic (*E* = −5068.2595 *E*_h_) and antiferromagnetic (*E* = −5068.2557 *E*_h_) coupling of the two Fe atoms in local d^5^ configurations (*S* = 5/2), as illustrated by singly occupied shell density plots in Figure 4B,C. These are the only two minima that were found with the open-shell electrons localized on only the Fe atoms, and the energy difference of 843.845 cm^–1^ matches previous results.^48^ In direct analogy with the mononuclear complex, the higher-energy local minima for [Fe_2_S_2_(SCH_3_)_4_]^2–^ correspond to electronic structures where one (or more) of the unpaired electrons is localized on a S atom. This result is illustrated using the singly occupied shell density plots for the first nonglobal minimum for each spin coupling in Figure 4B,C.

The increase in local minima and the larger spread of energies compared to the mononuclear complex can be explained by the larger number of S atoms in [Fe_2_S_2_(SCH_3_)_4_]^2–^. In particular, there are several ways to localize multiple unpaired electrons electrons onto different S environments, which leads to many nondegenerate CSF solutions. This result suggests that the number of local minima is likely to increase in larger complexes with more diverse atomic environments. Finally, despite the small number of samples, it is surprising that the random search did not find the antiferromagnetic ground state solution. These findings reinforce the importance of the initial guess in CSF optimization.

### 5.3 Open-Shell Character of Singlet Ground State in Polyacenes

Finally, the CSF approach can be used to qualitatively study the open-shell character in nonmetallic compounds, such as organic molecules. This approach can be illustrated using the singlet ground state in polyacene chains (C_4n+2_H_2n+4_), which are candidates for singlet fission materials^109,110^ or interstellar compounds,^111^ and provide building blocks for nanographene fragments.^112,113^ While high-accuracy wave function calculations,^114–117^ including DMRG,^16^ have shown that polyacenes have a singlet ground state, the qualitative open-shell character in large polyacenes is not as easy to deduce from correlated calculations. Spin-symmetry breaking in unrestricted KS-DFT has been proposed as evidence of a diradical ground state in larger polyacenes,^118^ while a second symmetry breaking in polyacenes with 13 or more rings indicates the onset of tetraradical character.^76^ The analysis of natural orbital occupation numbers from DMRG^16^ supports these findings, but it has been argued that the presence of symmetry breaking in KS-DFT is not a reliable diagnostic for open shell character due to the possibility of artificial symmetry breaking,^119^ which is common in SCF calculations with significant HF exchange.^74,75^

Optimising individual CSFs with different open-shell character provides an alternative route to probe the qualitative nature of the singlet ground state without resorting to spin-symmetry breaking, while retaining mean-field computational cost. In particular, the energies of CSF solutions with closed-shell, diradical, tetraradical, or hexaradical spin coupling can be compared to identify the most stable number of unpaired electrons in the ground state and where they are localized in the molecule. To test this idea, CSF-GDM calculations with various degrees of polyradical character were performed for the *n*-acene series up to *n* = 16 using the cc-pVDZ basis set.^120^ The largest calculation includes over 100 atoms and 1104 MOs, illustrating the scalability of CSF-GDM. Molecular structures for *n* = 2–6, 8, 10, and 12 were taken from ref 16., while structures for *n* = 7, 9, 11, and 13–16 were computed using UB3LYP/6-31G­(d) in Q-Chem^121^ (see Supporting Information Section S2). Initial coefficients were obtained from the high-spin ROHF orbitals, which were localized and assigned to shells using the algorithm outlined in Appendix B.

The energies of the ground-state CSF solutions with diradical, tetraradical, or hexaradical spin coupling are shown relative to the corresponding RHF solution for each structure in Figure 5A. Only the [+–+−] tetraradical and [+–+–+−] hexaradical solutions are shown, although the energetics are qualitatively unchanged if the other *S* = 0 spin coupling patterns with 4 or 6 unpaired electrons are used instead (see Supporting Information Table S5). For the short polyacenes, the closed-shell solution is lowest in energy and the ground state is expected to be dominated by closed-shell character. However, as the number of rings increases, the lowest-energy solution becomes the diradical CSF at 8-acene, and then becomes the tetradradical CSF for 15-acene. This transition in the lowest-energy CSF solution supports the onset of polyradical character for the longer polyacenes.

**Figure 5:**
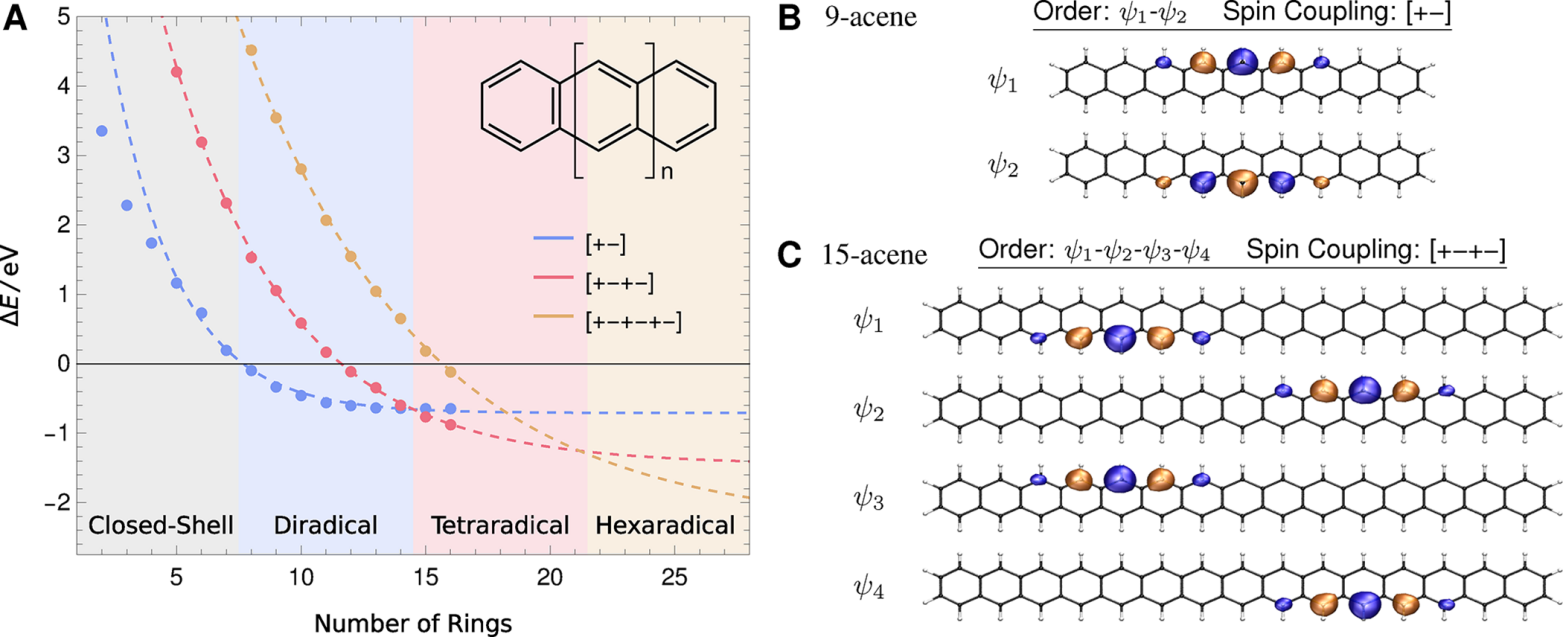
(A) Energies of the singlet ground state CSF solutions (cc-pVDZ) with diradical [+−], tetraradical [+–+−], and hexaradical [+–+–+−] spin coupling in polyacenes, plotted relative to the closed-shell RHF energy. Total energies are provided in Supporting Information Table S5. Changes in the lowest energy CSF indicate the onset of polyradical character for larger polyacenes. (B) Open-shell orbitals for the diradical CSF [+–−] in 9-acene. (**C**) Open-shell orbitals for the tetraradical CSF [+−] in 15-acene.

In ref 76, Trinquier and co-workers proposed that the longer acenes are composed of multiple disjoint diradicals localized in tetra-methylene units linked by aromatic napthalene or benzene rings. Their argument was motivated by the onset of KS-DFT spin symmetry breaking in 7-acene, with a second symmetry breaking at 13-acene. Remarkably, this qualitative picture is corroborated by the relative energies of the different CSF states, which suggest a transition to diradical character around 7–8 rings, and to tetraradical character around 14–15 rings. Extrapolation using an exponential fit (dashed line Figure 5A) suggests that the ground state becomes hexaradical at around 21–22 rings, which again matches the prediction in ref 76. Furthermore, the open-shell orbitals for the diradical CSF in 9-acene (Figure 5B) and the tetraradical CSF in 15-acene (Figure 5B) support the predicted localization of each unpaired electron to five carbons on one edge of the polyacene chain. Notably, these local orbitals emerge directly from the CSF optimization without any post-SCF localization, in contrast to the approach described in ref 76.

These results suggest that CSF calculations can provide valuable insights into the localization of unpaired electrons, giving a conceptual understanding of open-shell electronic structures. Assessing open-shell character using natural occupation numbers from correlated wave functions involves a degree of arbitrariness as the occupation number varies continuously from 0 to 2. On the other hand, unphysical “artificial” spin-symmetry breaking in KS-DFT can suggest open shell character in molecules that would be considered closed-shell, such as benzene.^66,74,75^ A single mean-field CSF provides an intermediate picture that retains spin symmetry, naturally results in localized unpaired electrons, and has fixed natural orbital occupations of 0, 1, or 2. Potential applications of this low-cost methodology might include graphene nanoflakes beyond the reach of active space studies, where unpaired electrons lead to small energy gaps and exotic magnetic properties.^113,122^

## 6 Conclusions

CSF-based ROHF theory is a promising approach to obtain compact reference states for open-shell problems without relying on CASSCF theory. However, finding the optimal orbitals for a given CSF requires new optimization algorithms for an arbitrary number of open shells. In this work, I have introduced a quasi-Newton Riemannian optimization algorithm “CSF-GDM” that enables robust energy minimization for CSFs with arbitrary genealogical spin coupling. This approach takes into account the structure of the CSF orbital constraint manifold and provides a generalized open-shell extension to the single-determinant GDM algorithm.^55^ Compared to the CSF-ROHF approach introduced recently in ref 48, the CSF-GDM algorithm generally converges in fewer iterations, is much less likely to find higher-energy local minima, and avoids saddle points of the energy.

Robust energy minimization using CSF-GDM has allowed important properties of CSF-based calculations to be investigated. Using the [Fe­(SCH_3_)_4_]^−^ and [Fe_2_S_2_(SCH_3_)_4_]^2–^ complexes as illustrative examples, I have shown that there can be many local minima on the CSF energy landscape, particularly for a CSF with several distinct open shells. These higher-energy solutions mimic key correlation processes, such as ligand-to-metal charge transfer, providing some qualitative insight into the true electronic structure of the ground state. However, the presence of many local minima emphasizes the importance of finding a good initial orbital guess that reflects the physical open-shell character and long-range electron correlation. More detailed studies into the electronic energy landscape of CSF-based ROHF theory will be required to fully characterize the properties and impact of these multiple solutions.

Furthermore, I have shown how mean-field CSF calculations can be used to gain qualitative insights into the localization of unpaired electrons in open-shell ground states. Comparing the relative energies of CSF solutions with different numbers of unpaired electrons revealed that the singlet ground state of polyacene chains becomes progressively polyradical as the number of rings increases, in line with previous predictions.^76^ The ability of CSF-based ROHF theory to provide spin-pure open-shell solutions, which automatically localize unpaired electrons where appropriate, creates a valuable new tool for studying polyradical molecules and magnetic compounds. Crucially, it does not rely on mean-field spin-symmetry breaking, which can be an unreliable indicator of open-shell character due to the possibility of artificial symmetry breaking.^63,66,74,75^

Moving forward, the ability to optimize low-spin open-shell configurations with an arbitrary number of unpaired electrons at mean-field cost creates several opportunities to develop new methodology. CSF states have already been successfully used to create minimal multiconfigurational reference states and a sparse Hilbert space for approximate CI calculations,^27–30,35,123^ and to define high-fidelity initial states for future quantum algorithms.^32,38,124,125^ In addition, it would be interesting to consider new single-reference correlation theories by applying many-body perturbation theory directly to an optimized CSF solution, providing a route toward quantitative energies for low-energy spin states, or ionization energies and electron affinities in open-shell systems.^126^

## Supplementary Material



## Data Availability

The data that supports the findings of this study are available within the article and its Supporting Information. All numerical data and molecular structures are available in a publicly available repository [10.5281/zenodo.16738382].

## Appendix A: Derivation of Analytic Hessian Terms

Following ref 26, the analytic second derivatives of the energy for an arbitrary wave function are given by

$$Q_{pqrs}=P_{pq}P_{rs}[2\gamma_{pr}h_{qs}-\delta_{qs}(F_{pr}^{P}+F_{rp}^{R})+2Y_{pqrs}]$$
A1

where *P*_*pq*_ = 1 – (*pq*) introduces an antisymmetric permutation of the indices *p* and *q*, and *F*_*pq*_ are the elements of the generalized Fock matrix defined in eq 20. The intermediate terms *Y*_*pqrs*_ are defined as

$$Y_{pqrs}=\sum_{mn}[(\Gamma_{prmn}+\Gamma_{pnmr})\langle qn|ms\rangle +\Gamma_{pmrn}\langle qm|sn\rangle ]$$
A2

Explicit expressions for the *Y*_*pqrs*_ terms can be obtained using the structure of the CSF density matrices. For a single CSF, the only nonzero 2-RDM terms are Γ_*pqpq*_ and Γ_*pqqp*_, leading to the expression

$$\begin{array}{rl} Y_{pqrs} & =\delta_{pr}\sum_{m}(\Gamma_{pmpm}\langle qm|sn\rangle +\Gamma_{pmmp}\langle qm|ms\rangle )\\ & +\delta_{pr}\Gamma_{pppp}\langle qp|ps\rangle +2(1-\delta_{pr})\Gamma_{prpr}\langle qr|ps\rangle \\ & +(1-\delta_{pr})\Gamma_{prrp}(\langle qp|rs\rangle +\langle qr|sp\rangle ). \end{array}$$
A3

Exploiting the relationships Γ_*pmmp*_ = Γ_*pmpm*_ when *p* = *m*, δ_*pr*_Γ_*prpr*_ ⟨*qr*|*ps*⟩ = Γ_*pppp*_ ⟨*qp*|*ps*⟩, and (1 – δ_*pr*_)­δ_*pr*_ = 0 gives

$$\begin{array}{rl} Y_{pqrs} & =\delta_{pr}\sum_{m}\Gamma_{pmpm}\langle qm|sn\rangle \\ & +\delta_{pr}\sum_{m}(\Gamma_{pmmp}-\delta_{pm}\Gamma_{pmpm})\langle qm|ms\rangle \\ & +2\Gamma_{prpr}\langle qr|ps\rangle \\ & +(\Gamma_{prrp}-\delta_{pr}\Gamma_{prpr})(\langle qp|rs\rangle +\langle qr|sp\rangle ), \end{array}$$
A4

where the (1 – δ_*pr*_) term on the last line of eq A3 is dropped by noting that (Γ_*prrp*_ – δ_*pr*_Γ_*prpr*_) = 0 when *p* = *r*. The coupling constants *a*_*pq*_ = Γ_*pqpq*_ and *b*_*pq*_ = Γ_*pqqp*_ – δ_*pq*_Γ_*pqpq*_ can now be inserted to obtain

$$\begin{array}{rl} Y_{pqrs} & =\delta_{pr}\sum_{m}(a_{pm}\langle qm|sm\rangle +b_{pm}\langle qm|ms\rangle )\\ & +2a_{pr}\langle qr|ps\rangle +b_{pr}(\langle qp|rs\rangle +\langle qr|sp\rangle ). \end{array}$$
A5

Using the definition of the Coulomb and exchange matrices in eq 21 allows this expression to be given as

$$\begin{array}{rl} Y_{pqrs} & =\delta_{pr}(n_{P}J_{qs}+K_{qs}^{P})\\ & +2a_{pr}\langle qr|ps\rangle +b_{pr}(\langle qp|rs\rangle +\langle qr|sp\rangle ). \end{array}$$
A6

The definition of the generalized Fock matrix elements in eq 20 then yields

$$\begin{array}{rl} Y_{pqrs} & =\delta_{pr}(F_{qs}^{P}-n_{p}h_{qs})\\ & +2a_{pr}\langle qr|ps\rangle +b_{pr}(\langle qp|rs\rangle +\langle qr|sp\rangle ). \end{array}$$
A7

Finally, substituting eq [Disp-formula eqa7] into eq [Disp-formula eqa1] and using $\gamma_{pr}=\delta_{pr}n_{P}$ yields the second derivatives in eq 24.

## Appendix B: Optimal Ordering of Open-Shell Orbitals

When initializing a CSF using localized orbitals, it is important that the orbitals are assigned to different open shells in a physically meaningful way. For example, in an antiferromagnetic bimetallic complex, the unpaired electrons on each metal center should occupy a shell with local ferromagnetic coupling, while the long-range coupling between the two metal centers should be antiferromagnetic. One approach to achieve the optimal assignment of initial local orbitals to each shell is to use combinatorial optimization to find the ordering that minimizes the exchange energy. While this task has previously been addressed using simulated annealing in ref 30, here I define a simple local search based on swapping two spatial orbitals at a time.

The starting point for this optimization is to note that the exchange energy for a CSF with *N*_o_ open-shell orbitals ordered as {ψ_1_, ψ_2_, ···, ψ_*N*_o__} is given by^30^

$$E_{x}^{\mathrm{os}}(\mu )=\sum_{w>v}b_{vw}K_{vw}(\mu )$$
B1

where the exchange integrals *K*_*vw*_(**μ**) = ⟨*vw*|*wv*⟩ depend on the ordering **μ** of the spatial orbitals, e.g., **μ** = (1–2-···-*N*_o_). Here, the indices *v*, *w* correspond to the orbital at a given position in **μ**. Only the energy contribution *E*_*x*_^os^(**μ**) is affected when the ordering of open-shell orbitals changes. Local steps in the combinatorial space of orbital orderings can be defined by permuting two spatial orbitals. For example, applying the permutation (12) to **μ** = (1–2-···-*N*_o_) gives (12) **μ** = (2–1-···-*N*_o_), which changes the energy if the orbitals in the first and second positions occupy different shells. The energy difference Δ*E*_*x*_^os^ for two orbital orderings **μ** and **μ** *′* can be computed as

$$\Delta E_{x}^{\mathrm{os}}=\sum_{w>v}b_{vw}(K_{vw}(\mu^{\prime })-K_{vw}(\mu ))$$
B2

Starting from a particular ordering **μ**_0_, a local downhill search can then be defined by systematically testing the change in *E*_*x*_^os^(**μ**) when two orbitals are swapped and accepting the swap if it lowers the energy. In practice, all possible swaps are considered sequentially, and the process is repeated until no further energy reduction can be achieved. While this approach is not guaranteed to find the globally optimal ordering of orbitals, it can significantly improve the initial guess for CSF optimization.

## Notes

Details of geometry optimization for the transition metal hexa-aquo complexes and polyacene chains, with optimized molecular coordinates, and converged CSF energies for all systems considered (PDF)
