# Supplementary material for: Geometric Direct Minimization for Low-Spin Restricted Open-Shell Hartree–Fock Theory
Source: J Chem Theory Comput. 2025 Sep 17;21(19):9444–58. doi: 10.1021/acs.jctc.5c00898 (PMC12529922; doi:10.1021/acs.jctc.5c00898)
Supplement: Supplementary file 1 [file ct5c00898_si_001.pdf]

Geometric direct minimization for low-spin restricted open-shell Hartree–Fock: Supplementary Material

Hugh G. A. Burton*
Department of Chemistry, University College London, London, WC1H 0AJ, U.K.
(Dated: September 10, 2025)

S1. GEOMETRY OPTIMIZATION FOR HEXA-AQUO COMPLEXES

Geometry optimization for the $[V(H_2O)_6]^{2+}$, $[Cr(H_2O)_6]^{3+}$, $[Mn(H_2O)_6]^{2+}$, $[Fe(H_2O)_6]^{3+}$, and $[Ni(H_2O)_6]^{2+}$ complexes was performed using ORCA 6.0.[1] The BP86 functional was used with the def2-SVP basis set and the def2/J auxiliary basis set was used to approximate the Coulomb integrals with the RI-J approach. The standard "tight" convergence criteria for geometry optimization were used. Converged geometries are listed below, with coordinates provided in angstroms, and were used for all subsequent calculations with the same metal centre regardless of the oxidation state or spin coupling.

S1.1. $[V(H_2O)_6]^{2+}$

```
V    0.000000    0.000000    0.000000
O    2.139821   -0.000001    0.000000
O   -2.139821    0.000001    0.000001
O   -0.000002    2.139534    0.000000
O    0.000002   -2.139534    0.000000
O    0.000000   -0.000001   -2.139594
O    0.000000    0.000000    2.139594
H    2.726238    0.783230    0.000000
H    2.726236   -0.783233    0.000000
H    0.000000    2.725892   -0.783273
H    0.000000    2.725892    0.783273
H   -0.783240    0.000001    2.726001
H    0.783241    0.000000    2.726001
H   -2.726237    0.783232    0.000001
H   -2.726238   -0.783231    0.000000
H    0.000000   -2.725891    0.783273
H    0.000000   -2.725892   -0.783273
H   -0.783241    0.000000   -2.726001
H    0.783240    0.000000   -2.726001
```

S1.2. $[Cr(H_2O)_6]^{3+}$

```
Cr   0.000000    0.000000    0.000000
O    2.002561    0.000000    0.000000
O   -2.002561    0.000000    0.000001
O   -0.000001    2.002561    0.000000
O    0.000000   -2.002561    0.000000
O    0.000000    0.000000   -2.002561
O    0.000000    0.000000    2.002561
H    2.584133    0.799039   -0.000001
H    2.584131   -0.799040    0.000000
H    0.000000    2.584132   -0.799039
H   -0.000001    2.584132    0.799039
H   -0.799039    0.000000    2.584132
```

* h.burton@ucl.ac.uk

H	0.799039	0.000000	2.584132
H	-2.584132	0.799040	0.000000
H	-2.584132	-0.799039	0.000001
H	0.000000	-2.584132	0.799039
H	0.000000	-2.584132	-0.799039
H	-0.799039	0.000000	-2.584132
H	0.799039	0.000000	-2.584132

S1.3. $[Mn(H_2O)_6]^{2+}$

Mn	0.000000	0.000000	0.000000
O	2.193894	-0.000001	0.000000
O	-2.193894	0.000001	0.000001
O	-0.000001	2.193820	0.000000
O	0.000001	-2.193820	0.000000
O	0.000000	0.000000	-2.193836
O	0.000000	0.000000	2.193836
H	2.785620	0.780200	0.000000
H	2.785618	-0.780203	0.000000
H	-0.000001	2.785543	-0.780204
H	-0.000001	2.785543	0.780203
H	-0.780204	0.000001	2.785559
H	0.780204	0.000000	2.785559
H	-2.785619	0.780203	0.000001
H	-2.785620	-0.780201	0.000001
H	0.000001	-2.785543	0.780203
H	0.000001	-2.785543	-0.780203
H	-0.780204	0.000000	-2.785559
H	0.780204	0.000000	-2.785559

S1.4. Optimized $[Fe(H_2O)_6]^{3+}$ geometry

Fe	0.000000	0.000000	0.000000
O	2.070726	0.000000	0.000000
O	-2.070726	0.000000	0.000000
O	0.000000	2.070719	0.000000
O	0.000000	-2.070719	0.000000
O	0.000000	0.000000	-2.070721
O	0.000000	0.000000	2.070721
H	2.665655	0.790685	-0.000001
H	2.665654	-0.790686	0.000000
H	-0.000001	2.665646	-0.790685
H	0.000000	2.665646	0.790684
H	-0.790685	0.000000	2.665648
H	0.790685	0.000000	2.665648
H	-2.665654	0.790686	0.000000
H	-2.665655	-0.790685	0.000000
H	0.000000	-2.665646	0.790685
H	0.000000	-2.665647	-0.790684
H	-0.790685	0.000001	-2.665648
H	0.790685	0.000000	-2.665648

S1.5. $[Ni(H_2O)_6]^{2+}$

Ni	0.000000	0.000000	0.000000

O	2.064359	0.000001	-0.000001
O	-2.064359	-0.000001	0.000001
O	-0.000004	2.064331	0.000000
O	0.000004	-2.064331	0.000000
O	0.000000	-0.000001	-2.064338
O	0.000000	0.000000	2.064338
H	2.644667	0.787697	0.000000
H	2.644666	-0.787696	0.000000
H	0.000003	2.644638	-0.787698
H	0.000002	2.644638	0.787698
H	-0.787697	0.000002	2.644644
H	0.787698	-0.000001	2.644644
H	-2.644667	0.787696	0.000000
H	-2.644667	-0.787697	0.000000
H	-0.000003	-2.644637	0.787698
H	-0.000002	-2.644637	-0.787698
H	-0.787698	0.000002	-2.644644
H	0.787698	-0.000001	-2.644644

S2. GEOMETRY OPTIMIZATION FOR POLYACENE CHAINS

Geometries for the polyacenes with 2–6, 8, 10, and 12 rings were taken from Ref. 2. The remaining structures were obtained through geometry optimization using the UB3LYP functional and the 6-13G(d) basis set with the Q-Chem package.[3] Stability analysis was performed to ensure that the UB3LYP energy corresponds to a local minimum, and the default convergence criteria for geometry optimization was used. The optimized structures are listed below, with coordinates provided in angstroms.

S2.1. 7-acene

C	8.598051	-0.713594	0.000000
C	8.598051	0.713594	0.000000
H	9.544277	1.247513	0.000000
H	9.544277	-1.247513	0.000000
C	7.416797	-1.407835	0.000000
C	7.416797	1.407835	0.000000
C	6.159793	-0.724824	0.000000
C	6.159793	0.724824	0.000000
H	7.414608	-2.495357	0.000000
H	7.414608	2.495357	0.000000
C	4.939989	-1.406488	0.000000
C	4.939989	1.406488	0.000000
C	3.704987	-0.728255	0.000000
C	3.704987	0.728255	0.000000
H	4.940732	-2.494698	0.000000
H	4.940732	2.494698	0.000000
C	2.472632	-1.409137	0.000000
C	2.472632	1.409137	0.000000
C	1.242656	-0.731128	0.000000
C	1.242656	0.731128	0.000000
H	2.473609	-2.497179	0.000000
H	2.473609	2.497179	0.000000
C	0.009742	-1.410171	0.000000
C	0.009742	1.410171	0.000000
C	-1.223114	-0.731155	0.000000
C	-1.223114	0.731155	0.000000
H	0.009789	-2.498177	0.000000

H	0.009789	2.498177	0.000000
C	-2.453118	-1.409158	0.000000
C	-2.453118	1.409158	0.000000
C	-3.685509	-0.728309	0.000000
C	-3.685509	0.728309	0.000000
H	-2.454064	-2.497200	0.000000
H	-2.454064	2.497200	0.000000
C	-4.920420	-1.406463	0.000000
C	-4.920420	1.406463	0.000000
C	-6.140395	-0.724878	0.000000
C	-6.140395	0.724878	0.000000
H	-4.921321	-2.494676	0.000000
H	-4.921321	2.494676	0.000000
C	-7.397199	-1.407872	0.000000
C	-7.397199	1.407872	0.000000
C	-8.578512	-0.713548	0.000000
C	-8.578512	0.713548	0.000000
H	-7.395399	-2.495429	0.000000
H	-7.395399	2.495429	0.000000
H	-9.525064	-1.247176	0.000000
H	-9.525064	1.247176	0.000000

S2.2. 9-acene

H	12.000646	-1.247010	0.000000
H	12.000646	1.247010	0.000000
C	11.054341	0.712944	0.000000
C	11.054341	-0.712944	0.000000
C	9.872162	1.407305	0.000000
C	9.872162	-1.407305	0.000000
C	8.616735	0.724027	0.000000
C	8.616735	-0.724027	0.000000
H	9.870303	2.494863	0.000000
H	9.870303	-2.494863	0.000000
C	7.394490	1.405726	0.000000
C	7.394490	-1.405726	0.000000
C	6.162823	0.727294	0.000000
C	6.162823	-0.727294	0.000000
H	7.395389	2.493951	0.000000
H	7.395389	-2.493951	0.000000
C	4.925792	1.408213	0.000000
C	4.925792	-1.408213	0.000000
C	3.701327	0.730369	0.000000
C	3.701327	-0.730369	0.000000
H	4.926851	2.496288	0.000000
H	4.926851	-2.496288	0.000000
C	2.461768	1.409364	0.000000
C	2.461768	-1.409364	0.000000
C	1.234362	0.731717	0.000000
C	1.234362	-0.731717	0.000000
H	2.462201	2.497416	0.000000
H	2.462201	-2.497416	0.000000
C	-0.000003	1.409586	0.000000
C	-0.000003	-1.409586	0.000000
C	-1.234368	0.731717	0.000000
C	-1.234368	-0.731717	0.000000
H	-0.000003	2.497638	0.000000

H	-0.000003	-2.497638	0.000000
C	-2.461774	1.409364	0.000000
C	-2.461774	-1.409364	0.000000
C	-3.701333	0.730369	0.000000
C	-3.701333	-0.730369	0.000000
H	-2.462207	2.497416	0.000000
H	-2.462207	-2.497416	0.000000
C	-4.925797	1.408214	0.000000
C	-4.925797	-1.408214	0.000000
C	-6.162828	0.727294	0.000000
C	-6.162828	-0.727294	0.000000
H	-4.926857	2.496289	0.000000
H	-4.926857	-2.496289	0.000000
C	-7.394495	1.405726	0.000000
C	-7.394495	-1.405726	0.000000
C	-8.616739	0.724027	0.000000
C	-8.616739	-0.724027	0.000000
H	-7.395394	2.493951	0.000000
H	-7.395394	-2.493951	0.000000
C	-9.872166	1.407306	0.000000
C	-9.872166	-1.407306	0.000000
C	-11.054345	0.712944	0.000000
C	-11.054345	-0.712944	0.000000
H	-9.870308	2.494863	0.000000
H	-9.870308	-2.494863	0.000000
H	-12.000651	1.247009	0.000000
H	-12.000651	-1.247009	0.000000

S2.3. 11-acene

H	14.470733	-1.247046	0.000000
H	14.470733	1.247046	0.000000
C	13.524261	0.713284	0.000000
C	13.524261	-0.713284	0.000000
C	12.342619	1.407546	0.000000
C	12.342619	-1.407546	0.000000
C	11.086521	0.724289	0.000000
C	11.086521	-0.724289	0.000000
H	12.340623	2.495103	0.000000
H	12.340623	-2.495103	0.000000
C	9.865235	1.405847	0.000000
C	9.865235	-1.405847	0.000000
C	8.632906	0.727261	0.000000
C	8.632906	-0.727261	0.000000
H	9.865881	2.494068	0.000000
H	9.865881	-2.494068	0.000000
C	7.396470	1.408128	0.000000
C	7.396470	-1.408128	0.000000
C	6.172491	0.730023	0.000000
C	6.172491	-0.730023	0.000000
H	7.397399	2.496210	0.000000
H	7.397399	-2.496210	0.000000
C	4.931826	1.409062	0.000000
C	4.931826	-1.409062	0.000000
C	3.707288	0.731180	0.000000
C	3.707288	-0.731180	0.000000
H	4.932280	2.497131	0.000000

H	4.932280	-2.497131	0.000000
C	2.468996	1.409010	0.000000
C	2.468996	-1.409010	0.000000
C	1.240270	0.731381	0.000000
C	1.240270	-0.731381	0.000000
H	2.469118	2.497098	0.000000
H	2.469118	-2.497098	0.000000
C	0.006588	1.408865	0.000000
C	0.006588	-1.408865	0.000000
C	-1.227069	0.731380	0.000000
C	-1.227069	-0.731380	0.000000
H	0.006593	2.496965	0.000000
H	0.006593	-2.496965	0.000000
C	-2.455818	1.409011	0.000000
C	-2.455818	-1.409011	0.000000
C	-3.694090	0.731182	0.000000
C	-3.694090	-0.731182	0.000000
H	-2.455923	2.497099	0.000000
H	-2.455923	-2.497099	0.000000
C	-4.918639	1.409066	0.000000
C	-4.918639	-1.409066	0.000000
C	-6.159305	0.730035	0.000000
C	-6.159305	-0.730035	0.000000
H	-4.919071	2.497135	0.000000
H	-4.919071	-2.497135	0.000000
C	-7.383267	1.408137	0.000000
C	-7.383267	-1.408137	0.000000
C	-8.619730	0.727273	0.000000
C	-8.619730	-0.727273	0.000000
H	-7.384206	2.496219	0.000000
H	-7.384206	-2.496219	0.000000
C	-9.852019	1.405843	0.000000
C	-9.852019	-1.405843	0.000000
C	-11.073340	0.724286	0.000000
C	-11.073340	-0.724286	0.000000
H	-9.852753	2.494069	0.000000
H	-9.852753	-2.494069	0.000000
C	-12.329416	1.407561	0.000000
C	-12.329416	-1.407561	0.000000
C	-13.511056	0.713250	0.000000
C	-13.511056	-0.713250	0.000000
H	-12.327495	2.495115	0.000000
H	-12.327495	-2.495115	0.000000
H	-14.457524	1.247027	0.000000
H	-14.457524	-1.247027	0.000000

S2.4. 13-acene

C	15.976660	-0.713365	0.000000
C	15.976660	0.713365	0.000000
H	16.923169	1.247063	0.000000
H	16.923169	-1.247063	0.000000
C	14.795141	-1.407662	0.000000
C	14.795141	1.407662	0.000000
C	13.538840	-0.724341	0.000000
C	13.538840	0.724341	0.000000
H	14.793290	2.495302	0.000000

H	14.793290	-2.495302	0.000000
C	12.318046	-1.405994	0.000000
C	12.318046	1.405994	0.000000
C	11.085325	-0.727400	0.000000
C	11.085325	0.727400	0.000000
H	12.318781	-2.494201	0.000000
H	12.318781	2.494201	0.000000
C	9.849434	-1.408234	0.000000
C	9.849434	1.408234	0.000000
C	8.624899	-0.730085	0.000000
C	8.624899	0.730085	0.000000
H	9.850333	-2.496313	0.000000
H	9.850333	2.496313	0.000000
C	7.384905	-1.409142	0.000000
C	7.384905	1.409142	0.000000
C	6.159983	-0.731072	0.000000
C	6.159983	0.731072	0.000000
H	7.385279	-2.497204	0.000000
H	7.385279	2.497204	0.000000
C	4.921911	-1.408959	0.000000
C	4.921911	1.408959	0.000000
C	3.693839	-0.730980	0.000000
C	3.693839	0.730980	0.000000
H	4.921928	-2.497045	0.000000
H	4.921928	2.497045	0.000000
C	2.458833	-1.408530	0.000000
C	2.458833	1.408530	0.000000
C	1.228062	-0.730702	0.000000
C	1.228062	0.730702	0.000000
H	2.458769	-2.496641	0.000000
H	2.458769	2.496641	0.000000
C	-0.004674	-1.408345	0.000000
C	-0.004674	1.408345	0.000000
C	-1.237398	-0.730702	0.000000
C	-1.237398	0.730702	0.000000
H	-0.004668	-2.496466	0.000000
H	-0.004668	2.496466	0.000000
C	-2.468181	-1.408529	0.000000
C	-2.468181	1.408529	0.000000
C	-3.703177	-0.730981	0.000000
C	-3.703177	0.730981	0.000000
H	-2.468106	-2.496640	0.000000
H	-2.468106	2.496640	0.000000
C	-4.931260	-1.408958	0.000000
C	-4.931260	1.408958	0.000000
C	-6.169327	-0.731074	0.000000
C	-6.169327	0.731074	0.000000
H	-4.931270	-2.497044	0.000000
H	-4.931270	2.497044	0.000000
C	-7.394254	-1.409141	0.000000
C	-7.394254	1.409141	0.000000
C	-8.634254	-0.730090	0.000000
C	-8.634254	0.730090	0.000000
H	-7.394629	-2.497203	0.000000
H	-7.394629	2.497203	0.000000
C	-9.858780	-1.408240	0.000000
C	-9.858780	1.408240	0.000000
C	-11.094677	-0.727383	0.000000

C	-11.094677	0.727383	0.000000
H	-9.859698	-2.496317	0.000000
H	-9.859698	2.496317	0.000000
C	-12.327367	-1.405942	0.000000
C	-12.327367	1.405942	0.000000
C	-13.548358	-0.724401	0.000000
C	-13.548358	0.724401	0.000000
H	-12.328100	-2.494164	0.000000
H	-12.328100	2.494164	0.000000
C	-14.804632	-1.407651	0.000000
C	-14.804632	1.407651	0.000000
C	-15.986127	-0.713341	0.000000
C	-15.986127	0.713341	0.000000
H	-14.802722	-2.495203	0.000000
H	-14.802722	2.495203	0.000000
H	-16.932633	-1.247048	0.000000
H	-16.932633	1.247048	0.000000

S2.5. 15-acene

C	18.435118	-0.713296	0.000000
C	18.435118	0.713296	0.000000
H	19.381694	1.246846	0.000000
H	19.381694	-1.246846	0.000000
C	17.253609	-1.407658	0.000000
C	17.253609	1.407658	0.000000
C	15.997551	-0.724369	0.000000
C	15.997551	0.724369	0.000000
H	17.251820	2.495313	0.000000
H	17.251820	-2.495313	0.000000
C	14.776315	-1.405643	0.000000
C	14.776315	1.405643	0.000000
C	13.543569	-0.727420	0.000000
C	13.543569	0.727420	0.000000
H	14.776900	2.493909	0.000000
H	14.776900	-2.493909	0.000000
C	12.307929	-1.408655	0.000000
C	12.307929	1.408655	0.000000
C	11.083339	-0.730281	0.000000
C	11.083339	0.730281	0.000000
H	12.308693	2.496749	0.000000
H	12.308693	-2.496749	0.000000
C	9.843441	-1.409139	0.000000
C	9.843441	1.409139	0.000000
C	8.618080	-0.731090	0.000000
C	8.618080	0.731090	0.000000
H	9.843344	-2.497151	0.000000
H	9.843344	2.497151	0.000000
C	7.380593	-1.409025	0.000000
C	7.380593	1.409025	0.000000
C	6.151868	-0.730973	0.000000
C	6.151868	0.730973	0.000000
H	7.380588	-2.497105	0.000000
H	7.380588	2.497105	0.000000
C	4.917547	-1.408565	0.000000
C	4.917547	1.408565	0.000000
C	3.686315	-0.730516	0.000000

C	3.686315	0.730516	0.000000
H	4.917411	-2.496670	0.000000
H	4.917411	2.496670	0.000000
C	2.453786	-1.408231	0.000000
C	2.453786	1.408231	0.000000
C	1.221687	-0.730212	0.000000
C	1.221687	0.730212	0.000000
H	2.453697	-2.496352	0.000000
H	2.453697	2.496352	0.000000
C	-0.010466	-1.408119	0.000000
C	-0.010466	1.408119	0.000000
C	-1.242568	-0.730214	0.000000
C	-1.242568	0.730214	0.000000
H	-0.010455	-2.496245	0.000000
H	-0.010455	2.496245	0.000000
C	-2.474720	-1.408232	0.000000
C	-2.474720	1.408232	0.000000
C	-3.707214	-0.730522	0.000000
C	-3.707214	0.730522	0.000000
H	-2.474610	-2.496353	0.000000
H	-2.474610	2.496353	0.000000
C	-4.938482	-1.408564	0.000000
C	-4.938482	1.408564	0.000000
C	-6.172800	-0.730978	0.000000
C	-6.172800	0.730978	0.000000
H	-4.938335	-2.496669	0.000000
H	-4.938335	2.496669	0.000000
C	-7.401528	-1.409023	0.000000
C	-7.401528	1.409023	0.000000
C	-8.639071	-0.731127	0.000000
C	-8.639071	0.731127	0.000000
H	-7.401502	-2.497104	0.000000
H	-7.401502	2.497104	0.000000
C	-9.864402	-1.409187	0.000000
C	-9.864402	1.409187	0.000000
C	-11.104191	-0.730134	0.000000
C	-11.104191	0.730134	0.000000
H	-9.864763	-2.497246	0.000000
H	-9.864763	2.497246	0.000000
C	-12.328838	-1.408261	0.000000
C	-12.328838	1.408261	0.000000
C	-13.564750	-0.727393	0.000000
C	-13.564750	0.727393	0.000000
H	-12.329756	-2.496336	0.000000
H	-12.329756	2.496336	0.000000
C	-14.797401	-1.405936	0.000000
C	-14.797401	1.405936	0.000000
C	-16.018477	-0.724379	0.000000
C	-16.018477	0.724379	0.000000
H	-14.798140	-2.494157	0.000000
H	-14.798140	2.494157	0.000000
C	-17.274688	-1.407631	0.000000
C	-17.274688	1.407631	0.000000
C	-18.456234	-0.713311	0.000000
C	-18.456234	0.713311	0.000000
H	-17.272782	-2.495181	0.000000
H	-17.272782	2.495181	0.000000
H	-19.402726	-1.247040	0.000000

H	-19.402726	1.247040	0.000000

S2.6. 16-acene

C	19.665641	-0.713322	0.000000
C	19.665641	0.713333	0.000000
H	20.612447	1.246530	0.000000
H	20.612446	-1.246521	0.000000
C	18.484215	-1.407877	0.000000
C	18.484220	1.407894	0.000000
C	17.228134	-0.724395	0.000000
C	17.228132	0.724418	0.000000
H	18.482297	2.495609	0.000000
H	18.482243	-2.495597	0.000000
C	16.006936	-1.405779	0.000000
C	16.006935	1.405805	0.000000
C	14.774037	-0.727327	0.000000
C	14.774036	0.727352	0.000000
H	16.007528	2.494130	0.000000
H	16.007527	-2.494104	0.000000
C	13.538075	-1.408304	0.000000
C	13.538071	1.408327	0.000000
C	12.313429	-0.730095	0.000000
C	12.313428	0.730113	0.000000
H	13.538501	2.496438	0.000000
H	13.538514	-2.496412	0.000000
C	11.073938	-1.409660	0.000000
C	11.073931	1.409671	0.000000
C	9.848506	-0.731348	0.000000
C	9.848505	0.731350	0.000000
H	11.073848	2.497718	0.000000
H	11.073874	-2.497703	0.000000
C	8.610872	-1.409098	0.000000
C	8.610865	1.409091	0.000000
C	7.381998	-0.730977	0.000000
C	7.381996	0.730963	0.000000
H	8.610089	-2.497128	0.000000
H	8.610075	2.497121	0.000000
C	6.147876	-1.408593	0.000000
C	6.147872	1.408575	0.000000
C	4.916332	-0.730573	0.000000
C	4.916331	0.730551	0.000000
H	6.147669	-2.496708	0.000000
H	6.147662	2.496690	0.000000
C	3.684178	-1.408303	0.000000
C	3.684176	1.408279	0.000000
C	2.451707	-0.730213	0.000000
C	2.451707	0.730187	0.000000
H	3.683984	-2.496424	0.000000
H	3.683980	2.496400	0.000000
C	1.219935	-1.408144	0.000000
C	1.219934	1.408118	0.000000
C	-0.012296	-0.730076	0.000000
C	-0.012296	0.730050	0.000000
H	1.219856	-2.496268	0.000000
H	1.219855	2.496242	0.000000
C	-1.244508	-1.408140	0.000000

C	-1.244507	1.408115	0.000000
C	-2.476293	-0.730198	0.000000
C	-2.476292	0.730174	0.000000
H	-1.244455	-2.496263	0.000000
H	-1.244453	2.496238	0.000000
C	-3.708754	-1.408288	0.000000
C	-3.708752	1.408266	0.000000
C	-4.940908	-0.730554	0.000000
C	-4.940906	0.730535	0.000000
H	-3.708591	-2.496406	0.000000
H	-3.708587	2.496384	0.000000
C	-6.172432	-1.408613	0.000000
C	-6.172429	1.408596	0.000000
C	-7.406539	-0.731011	0.000000
C	-7.406537	0.730998	0.000000
H	-6.172227	-2.496716	0.000000
H	-6.172221	2.496700	0.000000
C	-8.635396	-1.409051	0.000000
C	-8.635392	1.409041	0.000000
C	-9.872851	-0.731143	0.000000
C	-9.872849	0.731138	0.000000
H	-8.635307	-2.497131	0.000000
H	-8.635300	2.497122	0.000000
C	-11.098216	-1.409198	0.000000
C	-11.098212	1.409197	0.000000
C	-12.337985	-0.730136	0.000000
C	-12.337983	0.730139	0.000000
H	-11.098510	-2.497258	0.000000
H	-11.098501	2.497257	0.000000
C	-13.562624	-1.408262	0.000000
C	-13.562619	1.408270	0.000000
C	-14.798543	-0.727387	0.000000
C	-14.798541	0.727400	0.000000
H	-13.563480	-2.496338	0.000000
H	-13.563470	2.496345	0.000000
C	-16.031175	-1.405930	0.000000
C	-16.031170	1.405948	0.000000
C	-17.252262	-0.724364	0.000000
C	-17.252259	0.724385	0.000000
H	-16.031872	-2.494152	0.000000
H	-16.031862	2.494169	0.000000
C	-18.508461	-1.407615	0.000000
C	-18.508455	1.407642	0.000000
C	-19.690012	-0.713285	0.000000
C	-19.690010	0.713317	0.000000
H	-18.506533	-2.495166	0.000000
H	-18.506523	2.495193	0.000000
H	-20.636498	-1.247025	0.000000
H	-20.636493	1.247060	0.000000

S3. SUPPLEMENTARY RESULTS

TABLE S1: Converged CSF energies (E_h) for the hexa-aqua complexes (def2-SVP) using initial orbitals from a converged high-spin ROHF calculation. Solutions that are not the lowest energy for a given structure and spin coupling are highlighted in italics.

		CSF-GDM	CSF-GDM (no PC)	CSF-GDM (no PT)	L-BFGS	CSF-ROHF
$[V(H_2O)_6]^{2+}$	[++−]	−1398.232485	−1398.232485	−1398.232485	−1398.232485	*−1398.229444*
	[+−+]	−1398.232607	−1398.232607	−1398.232607	−1398.232607	*−1398.229424*
$[V(H_2O)_6]^{3+}$	[+−]	−1397.661357	−1397.661357	−1397.661357	−1397.661357	−1397.661357
$[Cr(H_2O)_6]^{2+}$	[++−−]	−1498.463166	−1498.463166	−1498.463166	−1498.463166	*−1498.460455*
	[+−+−]	−1498.476792	−1498.476792	−1498.476792	−1498.476792	*−1498.460483*
	[+++−]	−1498.511263	−1498.511263	−1498.511263	−1498.511263	−1498.511263
	[++−+]	−1498.516324	−1498.516324	−1498.516324	−1498.516324	*−1498.504505*
	[+−++]	−1498.521138	−1498.521138	−1498.521138	−1498.521138	*−1498.504351*
$[Cr(H_2O)_6]^{3+}$	[++−]	−1497.983320	−1497.983320	−1497.983320	−1497.983320	−1497.983320
	[+−+]	−1497.983502	−1497.983502	−1497.983502	−1497.983502	−1497.983502
$[Mn(H_2O)_6]^{2+}$	[+++−−]	−1604.961635	−1604.961635	−1604.961635	−1604.961635	−1604.961635
	[++−+−]	−1604.971353	−1604.971353	−1604.971353	−1604.971353	*−1604.920283*
	[+−++−]	−1604.977578	−1604.977578	−1604.977578	−1604.977578	*−1604.948127*
	[++−−+]	−1604.961448	−1604.961448	−1604.961448	−1604.961448	*−1604.958641*
	[+−+−+]	−1604.976065	−1604.976065	−1604.976065	−1604.976065	*−1604.930838*
	[++++−]	−1605.036258	−1605.036258	−1605.036258	−1605.036258	−1605.036258
	[+++−+]	−1605.040612	−1605.040612	−1605.040612	−1605.040612	failed
	[++−++]	−1605.047352	−1605.047352	−1605.047352	−1605.047352	*−1605.035037*
	[+−+++]	−1605.049050	−1605.049050	−1605.049050	−1605.049050	*−1605.034986*
$[Fe(H_2O)_6]^{2+}$	[++−−]	−1717.549602	−1717.549602	−1717.549602	−1717.549602	*−1717.546928*
	[+−+−]	−1717.564968	−1717.564968	−1717.564968	−1717.564968	*−1717.514983*
	[+++−]	−1717.606299	−1717.606299	−1717.606299	−1717.606299	*−1717.578786*
	[++−+]	−1717.613160	−1717.613160	−1717.613160	−1717.613160	−1717.613160
	[+−++]	−1717.616403	−1717.616403	−1717.616403	−1717.616403	*−1717.596583*
$[Fe(H_2O)_6]^{3+}$	[+++−−]	−1716.844253	−1716.844253	*−1716.843054*	−1716.844253	*−1716.770637*
	[++−+−]	−1716.920870	−1716.920870	−1716.920870	−1716.920870	−1716.920870
	[+−++−]	−1716.929605	−1716.929605	−1716.929605	−1716.929605	*−1716.872476*
	[++−−+]	−1716.935763	−1716.935763	−1716.935763	−1716.935763	*−1716.902139*
	[+−+−+]	−1716.916441	−1716.916441	−1716.916441	−1716.916441	*−1716.911895*
	[++++−]	−1716.933069	−1716.933069	−1716.933069	−1716.933069	*−1716.882275*
	[+++−+]	−1717.004821	−1717.004821	−1717.004821	−1717.004821	failed
	[++−++]	−1717.008644	−1717.008644	−1717.008644	−1717.008644	*−1717.008630*
	[+−+++]	−1717.013100	−1717.013100	−1717.013100	−1717.013100	*−1716.994885*
$[Ni(H_2O)_6]^{2+}$	[+−]	−1962.030449	*−1961.660731*	−1962.030449	−1962.030449	−1962.030447

TABLE S2: Converged CSF energies (E_h) for the hexa-aqua complexes (def2-SVP) starting from a core orbital guess. Solutions that are not the lowest energy for a given structure and spin coupling are highlighted in italics.

		CSF-GDM	CSF-GDM (no PC)	CSF-GDM (no PT)	L-BFGS	CSF-ROHF
$[V(H_2O)_6]^{2+}$	[++−]	−1398.232485	−1398.232485	−1398.232485	−1398.232485	*−1398.229444*
	[+−+]	−1398.232607	−1398.232607	−1398.232607	−1398.232607	*−1397.684588*
$[V(H_2O)_6]^{3+}$	[+−]	−1397.661360	*−1397.661357*	*−1397.661357*	*−1397.661357*	*−1397.616299*
$[Cr(H_2O)_6]^{2+}$	[++−−]	−1498.463166	−1498.463166	−1498.463166	−1498.463166	failed
	[+−+−]	−1498.476792	−1498.476792	−1498.476792	*−1498.252800*	failed
	[+++−]	*−1498.511263*	−1498.513816	*−1498.511263*	−1498.513816	*−1498.449669*
	[++−+]	−1498.516324	−1498.516324	−1498.516324	−1498.516324	*−1498.447991*
	[+−++]	−1498.521138	−1498.521138	−1498.407759	−1498.521138	*−1498.015221*
$[Cr(H_2O)_6]^{3+}$	[++−]	−1497.983320	−1497.983320	−1497.983320	−1497.983320	*−1497.860372*
	[+−+]	−1497.983502	−1497.983502	−1497.983502	−1497.983502	*−1497.860372*
$[Mn(H_2O)_6]^{2+}$	[+++−−]	*−1604.756201*	−1604.962747	−1604.962747	−1604.962747	*−1604.962290*
	[++−+−]	*−1604.688782*	−1604.971353	*−1604.691008*	*−1604.669074*	*−1604.920283*
	[+−++−]	*−1604.725349*	*−1604.688703*	*−1604.724346*	−1604.977678	*−1604.948127*
	[++−−+]	−1604.961448	*−1604.731984*	*−1604.681743*	*−1604.731858*	*−1604.960656*
	[+−+−+]	*−1604.676426*	*−1604.712436*	*−1604.674691*	*−1604.681037*	−1604.958166
	[++++−]	*−1604.741928*	*−1604.697188*	−1605.038799	−1605.038799	*−1605.036258*
	[+++−+]	*−1604.708976*	*−1604.705059*	−1605.042800	*−1604.708976*	failed
	[++−++]	*−1604.685920*	−1605.047352	−1605.047352	*−1604.380856*	*−1605.045298*
	[+−+++]	−1605.049050	−1605.049050	*−1604.725803*	−1605.049050	*−1604.604434*
$[Fe(H_2O)_6]^{2+}$	[++−−]	−1717.549602	*−1717.336750*	*−1717.285303*	*−1717.332764*	*−1717.549338*
	[+−+−]	−1717.564967	−1717.564967	−1717.564967	*−1717.315657*	failed
	[+++−]	*−1717.322709*	−1717.606299	−1717.606299	*−1717.306544*	*−1717.605466*
	[++−+]	−1717.613616	−1717.613616	−1717.613616	*−1717.320002*	*−1717.607195*
	[+−++]	−1717.616403	−1717.616403	−1717.616403	*−1717.314152*	failed
$[Fe(H_2O)_6]^{3+}$	[+++−−]	−1716.844253	−1716.844253	*−1716.843054*	−1716.844253	*−1716.770637*
	[++−+−]	−1716.874681	−1716.874682	*−1716.855765*	*−1716.855765*	*−1716.789468*
	[+−++−]	−1716.886530	*−1716.558499*	−1716.886530	*−1716.850881*	failed
	[++−−+]	−1716.870752	−1716.870751	−1716.870752	*−1716.582388*	*−1716.802605*
	[+−+−+]	−1716.873332	*−1716.873015*	*−1716.840773*	*−1716.575707*	failed
	[++++−]	−1716.890762	−1716.890762	−1716.890762	−1716.890762	failed
	[+++−+]	−1716.900813	−1716.900813	*−1716.587090*	*−1716.597354*	*−1716.807952*
	[++−++]	*−1716.595715*	*−1716.911609*	−1716.981844	*−1716.911978*	*−1716.822176*
	[+−+++]	−1717.014072	*−1716.602559*	−1717.014072	−1717.014072	failed
$[Ni(H_2O)_6]^{2+}$	[+−]	−1962.030449	*−1961.660731*	−1962.030449	−1962.030449	−1962.030447

TABLE S3: Converged CSF local minima (E_h) for the $[Fe(SCH_3)_4]^-$ complex (def2-TZVP) with different spin coupling vectors. Local minima were identified from 100 random starting points for each spin coupling vector, and thus these results are not expected to be exhaustive.

[+++++]	[++++−]	[+++−+]	[++−++]	[+−+++]	[+++−−]	[++−+−]	[++−−+]	[+−++−]	[+−+−+]
−3011.148027	−3011.074238	−3011.067138	−3011.055969	−3011.031649	−3010.961468	−3010.967074	−3010.952295	−3010.967813	−3010.954751
−3010.817802	−3010.971925	−3010.980204	−3010.991521	−3011.006582	−3010.959982	−3010.957373	−3010.910967	−3010.967094	−3010.954651
	−3010.964410	−3010.965897	−3010.989927	−3010.972206	−3010.955729	−3010.939598	−3010.812140	−3010.963395	−3010.954638
	−3010.812503	−3010.813150	−3010.971243	−3010.970167	−3010.928486	−3010.939018	−3010.797844	−3010.951562	−3010.936472
	−3010.788247	−3010.810009	−3010.806773	−3010.809986	−3010.927872	−3010.923474	−3010.794186	−3010.934726	−3010.936169
	−3010.784887	−3010.807222	−3010.806469	−3010.809619	−3010.812468	−3010.923474	−3010.793118	−3010.934241	−3010.924884
	−3010.783940	−3010.795574	−3010.805941	−3010.800403	−3010.811521	−3010.807820	−3010.792962	−3010.934183	−3010.797985
	−3010.779942	−3010.789515	−3010.794059	−3010.799407	−3010.795239	−3010.793786	−3010.779037	−3010.934056	−3010.797884
	−3010.779377	−3010.783029	−3010.779276	−3010.798940	−3010.793966	−3010.790807	−3010.778955	−3010.811399	−3010.797413
	−3010.768639	−3010.782447	−3010.778586	−3010.798760	−3010.792541	−3010.789981	−3010.777849	−3010.807779	−3010.797251
	−3010.768597	−3010.777858	−3010.777797	−3010.797610	−3010.792379	−3010.789558	−3010.774317	−3010.798418	−3010.795544
		−3010.775764	−3010.773131	−3010.780004	−3010.792379	−3010.787054	−3010.773772	−3010.797858	−3010.794592
			−3010.772939	−3010.778958	−3010.778540	−3010.781585	−3010.770331	−3010.797808	−3010.792078
				−3010.778580	−3010.774035	−3010.780604	−3010.769867	−3010.797207	−3010.778836
					−3010.769559	−3010.772729	−3010.752346	−3010.796220	−3010.778211
					−3010.758251	−3010.770183	−3010.752069	−3010.796137	−3010.778122
					−3010.756989	−3010.769684	−3010.741951	−3010.794986	−3010.778059
					−3010.752921	−3010.766241		−3010.794972	−3010.777518
					−3010.746118	−3010.756142		−3010.780966	−3010.776498
						−3010.750103		−3010.780224	−3010.760867
						−3010.740558		−3010.780076	−3010.756526
								−3010.777359	
								−3010.776232	
								−3010.776083	
								−3010.774821	
								−3010.774253	
								−3010.767467	

TABLE S4: Converged CSF local minima (E_h) for the $[Fe_2S_2(SCH_3)_4]^{2-}$ complex (def2-TZVP) with different spin coupling vectors. Local minima were identified from 100 random starting points for each spin coupling vector, and thus these results are not expected to be exhaustive. The lowest energy minimum for the [+++++−−−−−] CSF was found using a physically motivated initial guess.

[++++++++++]	[+++++−−−−−]
−5068.259547	−5068.255702
−5068.177090	−5068.116669
−5068.131590	−5068.017194
−5068.129961	−5068.016641
−5067.899013	−5068.013984
−5067.891197	−5067.958889
−5067.890807	−5067.926329
−5067.890349	−5067.915549
−5067.856266	−5067.915204
−5067.849373	−5067.913038
−5067.831287	−5067.912944
	−5067.898220
	−5067.898205
	−5067.880973
	−5067.880866
	−5067.880700
	−5067.880684
	−5067.880578
	−5067.855703
	−5067.851253
	−5067.851121
	−5067.850536
	−5067.843574
	−5067.840123
	−5067.837030
	−5067.820394
	−5067.817593
	−5067.813364
	−5067.808262
	−5067.779421
	−5067.775166
	−5067.750712
	−5067.748120
	−5067.746993
	−5067.743366
	−5067.741975
	−5067.740841
	−5067.737344
	−5067.736869
	−5067.727692
	−5067.716496
	−5067.713647
	−5067.712966
	−5067.711750
	−5067.709824
	−5067.702642
	−5067.690213
	−5067.689277
	−5067.688770
	−5067.687884
	−5067.685743
	−5067.684540
	−5067.674906
	−5067.673248
	−5067.671300
	−5067.671259
	−5067.660326

TABLE S5: Converged singlet CSF energies (E_h) for the polyacene chains (cc-pVDZ) with various spin coupling patterns. There is only a small difference between the energies of different CSFs with the same number of unpaired electrons.

	RHF	[+−]	[+−+−]	[++−−]	[+−+−+−]	[+−++−−]	[++−−+−]	[++−+−−]	[+++−−−]
2-acene	−383.383583	−383.259781	−383.083887	−383.082226	−382.903844	−382.913455	−382.913455	−382.905721	−382.906628
3-acene	−536.037093	−535.952807	−535.767128	−535.778801	−535.594626	−535.592382	−535.595408	−535.622700	−535.597025
4-acene	−688.686483	−688.622185	−688.472769	−688.471530	−688.280774	−688.281562	−688.281745	−688.280672	−688.279787
5-acene	−841.333548	−841.290332	−841.178556	−841.179320	−840.970027	−840.970202	−840.993109	−840.970633	−840.969856
6-acene	−993.977729	−993.950342	−993.859855	−993.860255	−993.657053	−993.656555	−993.656236	−993.655825	−993.655424
7-acene	−1146.618183	−1146.610595	−1146.532579	−1146.533359	−1146.413158	−1146.414972	−1146.414972	−1146.415509	−1146.415579
8-acene	−1299.259619	−1299.262734	−1299.202943	−1299.200197	−1299.093123	−1299.093747	−1299.094806	−1299.095246	−1299.095440
9-acene	−1451.901606	−1451.913462	−1451.862300	−1451.861945	−1451.770908	−1451.771453	−1451.772303	−1451.771955	−1451.772797
10-acene	−1604.544206	−1604.560529	−1604.522174	−1604.518522	−1604.440539	−1604.440992	−1604.441976	−1604.442446	−1604.442649
11-acene	−1757.187011	−1757.207126	−1757.180353	−1757.181078	−1757.110486	−1757.111964	−1757.111964	−1757.112595	−1757.112816
12-acene	−1909.829803	−1909.851514	−1909.833500	−1909.833924	−1909.772490	−1909.773791	−1909.773791	−1909.774430	−1909.773884
13-acene	−2062.472532	−2062.495484	−2062.484845	−2062.485491	−2062.433782	−2062.434015	−2062.434820	−2062.435276	−2062.435406
14-acene	−2215.115105	−2215.138320	−2215.136672	−2215.136957	−2215.090703	−2215.090903	−2215.091992	−2215.092715	−2215.092872
15-acene	−2367.757749	−2367.781149	−2367.785453	−2367.785516	−2367.750564	−2367.750704	−2367.751521	−2367.751516	−2367.752145
16-acene	−2520.400348	−2520.423655	−2520.432267	−2520.432219	−2520.404177	−2520.404282	−2520.404973	−2520.404958	−2520.405478

REFERENCES

[1] Neese, F. Software update: The ORCA program system - Version 5.0. *WIREs Comput. Mol. Sci.* **2022**, *12*, e1606.
[2] Hachmann, J.; Dorando, J. J.; Avilés, M.; Chan, G. K.-L. The radical character of the acenes: A density matrix renormalization group study. *J. Chem. Phys.* **2007**, *127*, 134309.
[3] Shao, Y. et al. Advances in molecular quantum chemistry contained in the Q-Chem 4 program package. *Mol. Phys.* **2015**, *113*, 184.
